# The Transcription Factor C/EBP delta Has Anti-Apoptotic and Anti-Inflammatory Roles in Pancreatic Beta Cells

**DOI:** 10.1371/journal.pone.0031062

**Published:** 2012-02-08

**Authors:** Fabrice Moore, Izortze Santin, Tatiane C. Nogueira, Esteban N. Gurzov, Lorella Marselli, Piero Marchetti, Decio L. Eizirik

**Affiliations:** 1 Laboratory of Experimental Medicine, Université Libre de Bruxelles, Brussels, Belgium; 2 Metabolic Unit, Department of Endocrinology and Metabolism, University of Pisa, Pisa, Italy; Wayne State University, United States of America

## Abstract

In the course of Type 1 diabetes pro-inflammatory cytokines (e.g., IL-1β, IFN-γ and TNF-α) produced by islet-infiltrating immune cells modify expression of key gene networks in β-cells, leading to local inflammation and β-cell apoptosis. Most known cytokine-induced transcription factors have pro-apoptotic effects, and little is known regarding “protective” transcription factors. To this end, we presently evaluated the role of the transcription factor CCAAT/enhancer binding protein delta (C/EBPδ) on β-cell apoptosis and production of inflammatory mediators in the rat insulinoma INS-1E cells, in purified primary rat β-cells and in human islets. C/EBPδ is expressed and up-regulated in response to the cytokines IL-1β and IFN-γ in rat β-cells and human islets. Small interfering RNA-mediated C/EBPδ silencing exacerbated IL-1β+IFN-γ-induced caspase 9 and 3 cleavage and apoptosis in these cells. C/EBPδ deficiency increased the up-regulation of the transcription factor CHOP in response to cytokines, enhancing expression of the pro-apoptotic Bcl-2 family member BIM. Interfering with C/EBPδ and CHOP or C/EBPδ and BIM in double knockdown approaches abrogated the exacerbating effects of C/EBPδ deficiency on cytokine-induced β-cell apoptosis, while C/EBPδ overexpression inhibited BIM expression and partially protected β-cells against IL-1β+IFN-γ-induced apoptosis. Furthermore, C/EBPδ silencing boosted cytokine-induced production of the chemokines CXCL1, 9, 10 and CCL20 in β-cells by hampering IRF-1 up-regulation and increasing STAT1 activation in response to cytokines. These observations identify a novel function of C/EBPδ as a modulatory transcription factor that inhibits the pro-apoptotic and pro-inflammatory gene networks activated by cytokines in pancreatic β-cells.

## Introduction

Type 1 diabetes (T1D) is a multi-factorial disease where a chronic autoimmune assault results in a progressive β-cell loss and increased circulating blood glucose levels [1], [2]. The recent discovery of numerous T1D-associated susceptibly genes [3], [4], as well as T1D-predisposing environmental factors [5], [6], added new layers of complexity to our understanding of the disease. Pancreatic islet infiltration by activated immune cells and the development of an aberrant islet inflammation (insulitis) are assumed to represent common events in early T1D [1], [2], [7]. A detailed understanding of early insulitis, during which infiltrating autoimmune cells induce β-cell apoptosis and inflammation [1], [8], may indicate novel and rational approaches for therapeutic interventions [9]–[11].

The pro-inflammatory cytokines interleukin(IL)-1β, interferon(IFN)-γ and tumor necrosis factor(TNF)-α produced by infiltrating immune cells play a critical role in the progression of β-cell demise and apoptosis in T1D [1], [8], [12]–[14]. We previously demonstrated that these pro-inflammatory cytokines activate the transcription factors NF-κB, STAT1 and IRF-1 in β-cells, and performed a series of microarray analysis to determine the gene networks regulated by these transcription factors in β-cells [13], [15], [16]. Down-regulated genes targeted by the pro-inflammatory cytokines and regulated by NF-κB/STAT1 include genes associated with β-cell differentiation (e.g. *MafA*, *Pdx1*, *Isl1* and *Nkx2.2*) and function (e.g. insulin, Glut2, glucokinase, proconvertase 1 and 3) [13], [16]. On the other hand there is up-regulation of genes implicated in endoplasmic reticulum(ER)-stress, e.g. *XBP-1s*, *ATF6* and *CHOP* (*GADD153, Ddit3, C/EBPζ*) [17]–[19], and of inflammation-promoting genes, such as the CXC chemokines CXCL1, 9, 10 and the CC chemokines CCL2, 5 and 20 [16], [20]. β-cell production of chemokines may play a key role in the initiation [21] and persistence of insulitis in murine and human diabetes [1], [22]. Cytokine-induced chemokine production is tightly regulated by STAT1 in β-cells, while its downstream transcription factor IRF-1 exerts inhibitory effects on chemokine production through activation of the negative regulator SOCS-1 [16]. Combinations of IL-1β or TNF-α with IFN-γ also induce a major shift in the balance between the pro- and anti-apoptotic members of the apoptosis-regulating genes B cell lymphoma 2 (Bcl-2) family in β-cells [23], with Death Protein 5 (DP5) [24], BCL2 binding component 3 (BBC3/PUMA) [25] and BCL2-like 11 (BIM) [26], [27] contributing to cytokine-induced β-cells apoptosis. Cytokine-induced β-cell apoptosis occurs through the “intrinsic” mitochondrial pathway, involving translocation of the pro-apoptotic protein Bax to the mitochondria, depolarization of the mitochondrial membrane, cytochrome *c* release and activation of caspases 9 and 3 [23].

Further analysis of our microarray data pointed out to an early induction of the transcription factor CCAAT/enhancer binding protein delta (C/EBPδ) in cytokine-treated β-cell via NF-κB and STAT1 activation [13], [15], [16]. The role for this transcription factor in β-cell, however, remains to be clarified. The C/EBP family consists of six transcription factors (α, β, γ, δ, ε and ζ) sharing a highly conserved basic leucin zipper domain at the C-terminal region of the protein; this domain is involved in homo- or hetero-dimerization and in DNA binding activity [28]. C/EBPδ expression is induced in other cell types in response to various stimuli, including mitogens, hormones, toxins and cytokines (IL-1β, IL-6, IFN-γ), and is mostly regulated at the transcriptional level [28]. Unlike C/EBPα, β and ε that exist as different splicing variants displaying diverse functions [29], [30], only one C/EBPδ isoform has been identified in rodents and humans [28]. C/EBPδ dimerises with several members of the C/EPB family (α, β and ζ) but also with NF-κB1 p50, RelA, and the Ets family member PU.1. [31]–[34], allowing it to exert various functions in different cell types. C/EBPδ activities have been associated with adipocytes differentiation [35], learning and memory processes in neurons [36], tumor suppressor activities in mammary gland epithelial cells [37], [38] and with Toll-like Receptor-mediated production of pro-inflammatory cytokines in macrophages [39], but much less is known about this transcription factor as compared to other members of the C/EBP family [28].

We presently report that C/EBPδ is expressed in rat insulinoma cells, primary rat β-cells and human islets, and that its expression is up-regulated upon exposure to IL-1β+IFN-γ. Using several single and combined siRNA-mediated knockdown approaches, we demonstrate that C/EBPδ deficiency exacerbates cytokine-induced β-cell demise by promoting pro-apoptotic and pro-inflammatory signalling pathways. Likewise, C/EBPδ overexpression partially protects β-cells against cytokine-induced apoptosis. All together, these results identify C/EBPδ as a new transcription factor that exerts unique and non-redundant functions in β-cells by negatively regulating the deleterious effects of pro-inflammatory cytokines.

## Results

### The cytokines IL-1β and IFN-γ up-regulate C/EBPδ mRNA and protein expression in INS-1E cells, primary rat β-cells and human islets

**Figure 1:**
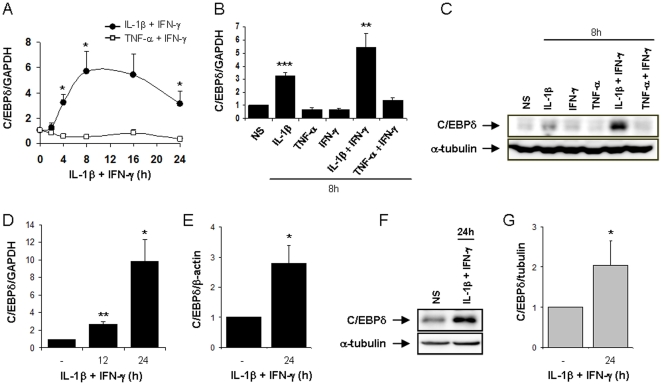
IL-1β and IFN-γ up-regulate C/EBPδ expression in INS-1E cells, primary rat β-cells and human islets. INS-1E cells (A–C), primary purified rat β-cells (D) or human islets (E–G) were left untreated or treated with either IL-1β, IFN-γ, TNF-α, IL-1β+IFN-γ or TNF-α+IFN-γ for the indicated time points. (A, B, D, E) C/EBPδ mRNA expression was assayed by RT-PCR and normalized for the housekeeping gene GAPDH (rat) or β-actin (human); (C, F) C/EBPδ and α-tubulin expressions were evaluated by Western blot. A representative experiment of 5–6 independent experiments is shown. (G) Mean optical density measurements of C/EBPδ Western blots corrected for protein loading by α-tubulin (representative figure in F). Results are mean ± SEM of 4–6 independent experiments; * *p*<0.05, ** *p*<0.01 and *** *p*<0.001 vs untreated cells by Student's *t* test.

Our previous microarray data suggest that C/EBPδ expression is modified by cytokine treatment in rat β-cells [13], [15], [16], [40]. In order to validate these observations, we evaluated C/EBPδ mRNA and protein expression in the rat insulinoma INS-1E cells, in primary FACS-sorted rat β-cells and human islets exposed to cytokines. C/EBPδ mRNA expression was up-regulated already after 4 h of IL-1β+IFN-γ treatment in INS-1E cells, reaching a peak at 8 h (6-fold increase) and remaining increased until 24 h. On the other hand, the combination of TNF-α+IFN-γ did not induce C/EBPδ expression at any of the tested time points (Figure 1A). Treatment of INS-1E cells for 8 h with each individual cytokine identified IL-1β as the key inducer of C/EBPδ expression; IFN-γ had no effect on its own, but it amplified IL-1β-induced C/EBPδ up-regulation (Figure 1B). Western blot analysis in INS-1E cells corroborated these observations, since C/EBPδ proteins were up-regulated by respectively 2- and 5-fold after 8 h of IL-1β and IL-1β+IFN-γ exposure (Figure 1C; densitometry in Figure S1A). These observations were confirmed in FACS-purified primary rat β-cells, in which IL-1β+IFN-γ induced a 3- and 10-fold up-regulation of C/EBPδ expression after 12 and 24 h respectively (Figure 1D) while its expression was unaffected by TNF-α+IFN-γ treatment (Figure S1B). In human islet cells, a 24 h exposure to IL-1β+IFN-γ up-regulated both C/EBPδ mRNA and protein expressions by 2–3 fold (Figure 1E–G). We also confirmed the role of the transcription factors NF-κB and STAT1 in cytokine-induced C/EBPδ up-regulation in β-cells [15], [16]. Thus, NF-κB blockade using a super-repressor IκBα inhibited IL-1β+IFN-γ-induced C/EBPδ transcription until 24 h (Figure S1C), while siRNA-mediated STAT1 knockdown affected mostly the early (12 h) up-regulation of C/EBPδ by cytokines (Figure S1D).

### siRNA-mediated C/EBPδ silencing exacerbates cytokine-induced apoptosis in INS-1E cells, primary rat β-cells, and dispersed human islets

**Figure 2:**
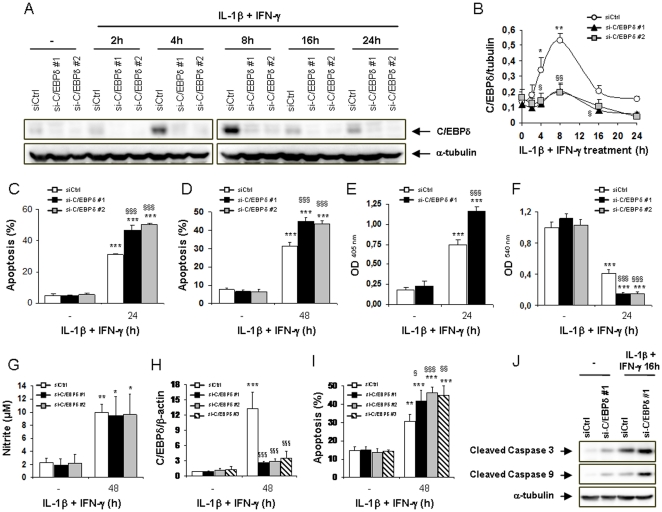
C/EBPδ silencing exacerbates cytokine-induced apoptosis in rat β-cells and human islets. (A, B, C, E, F) INS-1E cells were transfected with either a control siRNA (siCtrl – white dots/bars), or two differents siRNAs targeting C/EBPδ (siC/EBPδ #1 – black triangles/bars and siC/EBPδ #2 – grey squares/bars). Cells were then left untreated, or treated with IL-1β+IFN-γ as indicated; (A) C/EBPδ and α-tubulin expressions were evaluated by Western blot; (B) Mean optical density measurements of C/EBPδ Western blots corrected for α-tubulin (representative figure in A); (C) Apoptosis was assessed by HO/PI staining; (E) Apoptosis was evaluated using the Cell Death Detection ELISAplus kit; (F) Cell viability was evaluated using the neutral red-based toxicology kit. (D, G) Primary FACS-sorted rat β-cells were transfected as described above and subsequently left untreated or treated with IL-1β+IFN-γ as indicated; (D) Apoptosis was assessed by HO/PI staining. (G) Nitrite concentrations in supernatants were measured as described in Methods. (H–I) Dispersed human islets were transfected with siCtrl (white bars) or human siC/EBPδ #1 (black bars) or #2 (grey bars) or #3 (hatched bars) and subsequently treated with IL-1β+IFN-γ for 48 h; (H) C/EBPδ mRNA expression was assayed by RT-PCR and normalized for the housekeeping gene β-actin; (I) Apoptosis was assessed by HO/PI staining. (J) INS-1E cells were transfected with siCtrl or siC/EBPδ #1; Cleaved caspase 3, 9 and α-tubulin expressions were evaluated by Western blot. Results are mean ± SEM of 4–7 experiments; *: *p*<0.05, **: *p*<0.01 and ***: *p*<0.001 vs untreated transfected with the same siRNA; §: *p*<0.05, §§: *p*<0.01 and §§§: *p*<0.001 vs siCtrl treated with cytokines at the same time point; ANOVA followed by Student's *t* test with Bonferroni correction.

We next used a siRNA-mediated knockdown approach to evaluate a putative role for C/EBPδ in IL-1β+IFN-γ-induced β-cell apoptosis. INS-1E cells were transfected with an irrelevant control siRNA (siCtrl) or with two siRNAs targeting C/EBPδ (siC/EBPδ #1 and #2). Cells were subsequently left untreated, or treated for 2 to 24 h with IL-1β+IFN-γ. Cytokines induced C/EBPδ protein up-regulation, reaching a peak at 8 h and slowly decreasing until 24 h (Figure 2A and 2B). The two siRNAs targeting C/EBPδ accurately silenced its expression in both untreated and IL-1β+IFN-γ-treated cells (Figure 2A and 2B). As previously reported [8], [16], treatment with IL-1β+IFN-γ induced apoptosis in siCtrl-transfected INS-1E cells after 24 h and in primary β-cells after 48 h (Figure 2C & 2D), while inducing necrosis in few cells under all experimental conditions tested (<2% necrotic cells - data not shown). C/EBPδ silencing with the two siRNAs exacerbated INS-1E cell and primary β-cell apoptosis after exposure to IL-1β+IFN-γ (Figure 2C & 2D). This increased apoptosis was confirmed in INS-1E cells by a second method that detects cytoplasmic fragmented DNA (Figure 2E). In line with these observations, quantification of the remaining living cells by neutral red confirmed that C/EBPδ deficiency hampered the ability of the cells to survive the IL-1β+IFN-γ treatment (Figure 2F). On the other hand, C/EBPδ mRNA silencing did not induce apoptosis in untreated INS-1E cells or after treatments with IL-1β or IFN-γ alone (Figure S2A), and it neither exacerbated apoptosis (Figure S2B) nor decreased the survival (Figure S2C) of INS-1E cells exposed to the combination of TNF-α+IFN-γ. The enhanced apoptosis in IL-1β+IFN-γ-treated C/EBPδ-silenced β-cells could not be explained by a higher production of nitric oxide (NO), since nitrite was similarly produced by siCtrl- and siC/EBPδ-transfected cells after IL-1β+IFN-γ exposure in primary rat β-cells (Figure 2G) and INS-1E cells (Figure S2D). Furthermore, the production of intracellular reactive oxygen- or nitrogen species (ROS or RNS) was also similar in cytokine-treated siCtrl- and siC/EBPδ-transfected INS-1E cells (Figure S2E). These data suggest that the increased cytokine-induced apoptosis in C/EBPδ-silenced cells is probably not due to an amplified oxidative/nitrosative stress. Viability assays in dispersed human islet cells also demonstrated that efficient silencing of C/EBPδ with three different siRNAs (Figure 2H & Figure S2F) resulted in increased apoptotic cell death after 48 h of IL-1β+IFN-γ exposure (Figure 2I). Cytokine-triggered β-cell apoptosis occurs through the mitochondrial-dependent intrinsic pathway of cell death, involving the translocation of the pro-apoptotic protein Bax to the mitochondria, cytochrome *c* release from the mitochondria to the cytoplasm, and subsequent cleavage and activation of caspases 9 and 3 [17], [23], [24]. As shown in Figure S2G, IL-1β+IFN-γ-induced apoptosis in C/EBPδ-silenced cells followed the same pathway, with Bax staining showing strong co-localization with the mitochondrial marker ATP synthase in apoptotic cells (arrow), while it was weak and diffuse in living cells (Figure S2G). Moreover, cytochrome *c* staining co-localized with the mitochondrial marker AIF in siC/EBPδ-transfected living cells, while apoptotic cells had diffuse cytoplasmic cytochrome *c* staining (arrow), suggesting cytochrome *c* release from the mitochondria (Figure S2H). Western blot analysis demonstrated that cytokine-induced expression of cleaved caspase 9 and caspase 3 were enhanced in siC/EBPδ-transfected cells as compared to siCtrl-transfected counterparts (Figure 2J – densitometries are shown in Figures S2I & S2J), supporting the observations from the viability assays (Figure 2C & 2D) and confirming the involvement of the mitochondrial pathway. We next performed glucose oxidation tests to evaluate the putative role of C/EBPδ on the functional inhibition of β-cells induced by cytokines [8], [13], [16]. Cytokine treatment greatly reduced the ability of β-cells to oxidize glucose (Figure S2K). The inhibition of glucose oxidation was, however, similar in cytokine-treated siCtrl- and siC/EBPδ-transfected cells (Figure S2K), suggesting that C/EBPδ is not involved in inhibition of β-cell function.

We also evaluated a putative role of C/EBPβ in cytokine-induced β-cell death. This other member of the C/EBP family was previously shown to be up-regulated by cytokines in array analysis of cytokine-treated β-cells [13], [16] and to modulate mouse β-cell susceptibility to ER stress [41]. The expression of C/EBPβ was up-regulated in INS-1E and primary β-cells after 12–24 h of IL-1β+IFN-γ exposure (Figure S3A) [13], [16]. A 36–60% silencing of C/EBPβ using two different siRNAs (Figure S3B) did not affect cytokine-induced apoptosis in INS-1E cells (Figure S3C) while it slightly decreased NO production (Figure S3D).

### C/EBPδ knockdown enhances cytokine-induced CHOP expression and transcriptional activity in β-cells

**Figure 3:**
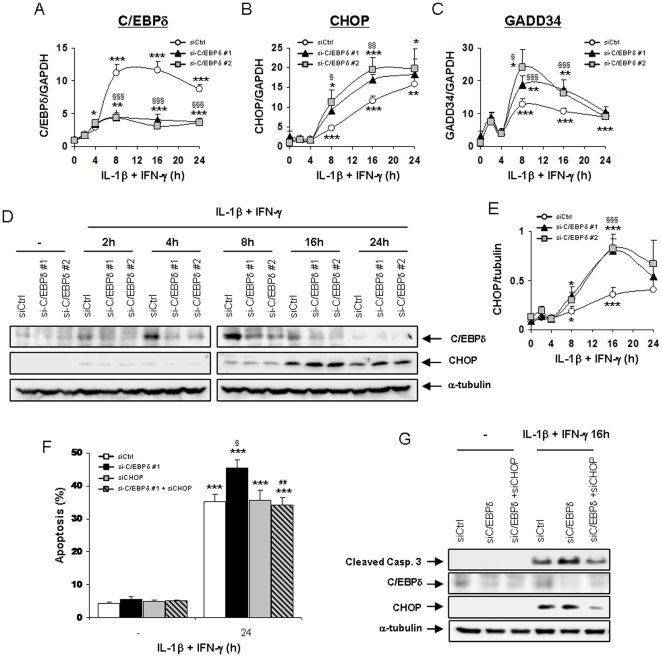
Amplified CHOP expression contributes to the exacerbation of apoptosis in cytokine-treated C/EBPδ-deficient INS-1E cells. (A–E) INS-1E cells were transfected with siCtrl (white dots), siC/EBPδ #1 (black triangles) or siC/EBPδ #2 (grey squares) and subsequently left untreated, or treated with IL-1β+IFN-γ for the indicated time points. (A–C) C/EBPδ, CHOP and GADD34 mRNA expressions were assayed by RT-PCR and normalized for the housekeeping gene GAPDH; (D) C/EBPδ, CHOP and α-tubulin expressions were evaluated by Western blot. (E) Mean optical density measurements of CHOP Western blots corrected for α-tubulin (representative figure in D). (F–G) INS-1E cells were transfected with siCtrl (white bars), siC/EBPδ #1 (black bars), siCHOP (grey bars) or siC/EBPδ #1+siCHOP (hatched grey bars) and subsequently left untreated, or treated with IL-1β+IFN-γ for 24 h as indicated. (F) Apoptosis was assessed by HO/PI staining. (G) Cleaved caspase 3, C/EBPδ, CHOP and α-tubulin expressions were evaluated by Western blot. Results are mean ± SEM of 4–5 experiments; *: *p*<0.05, **: *p*<0.01 and ***: *p*<0.001 vs untreated transfected with the same siRNA; §: *p*<0.05, §§: *p*<0.01 and §§§: *p*<0.001 vs siCtrl treated with cytokines at the same time point; ##: *p*<0.01 vs siC/EBPδ #1 treated with cytokines at the same time point; ANOVA followed by Student's *t* test with Bonferroni correction.

To clarify the molecular pathways underlying the exacerbation of cytokine-induced apoptosis in C/EBPδ-silenced cells, we first evaluated the expression of several genes induced during ER stress, a cellular response associated with IL-1β+IFN-γ-induced apoptosis in β-cells [18], [42]. XBP-1s mRNA expression was similarly up-regulated by cytokine treatment in siCtrl- and siC/EBPδ-transfected INS-1E cells and C/EBPδ silencing did not induce nuclear translocation of XBP-1s in cytokine-treated INS-1E cells (data not shown). Cytokine treatment equally decreased Bip mRNA expression at 24 h in the three transfected conditions. On the other hand, C/EBPδ deficiency enhanced IL-1β+IFN-γ-induced CHOP mRNA up-regulation after 8- and 16 h in INS-1E cells (Figure 3A & 3B). This increased CHOP expression was confirmed at the protein level after 16- and 24 h of cytokine treatment (Figure 3D & 3E) and had a functional impact, since the expression of the CHOP-dependent gene GADD34 was also augmented by C/EBPδ silencing in INS-1E cells (Figure 3C). Since CHOP is a member of the C/EBP family (C/EBPζ) [28] and may hetero-dimerize with C/EBPδ [43], we next evaluated a putative role of CHOP in the exacerbation of cytokine-induced apoptosis in C/EBPδ-silenced cells. To this end, we interfered in parallel with C/EBPδ and CHOP expressions, in a double knockdown approach, and performed viability assays. As described above, C/EBPδ deficiency exacerbated IL-1β+IFN-γ-induced apoptosis in INS-1E cells, while CHOP silencing had no effect on cell apoptosis after 24 h of cytokine treatment (Figure 3F). Interestingly, the concomitant knockdown of CHOP abrogated the exacerbating effect of C/EBPδ silencing on apoptosis (Figure 3F). These results were confirmed by Western blot for cleaved caspase 3: the simultaneous inhibition of C/EBPδ and CHOP reversed the increased expression of cleaved caspase 3 observed after C/EBPδ silencing (Figure 3G). These results identify CHOP as a contributory factor in the exacerbation of cytokine-induced apoptosis in C/EBPδ-silenced β-cells. Since CHOP may also regulate the mitochondrial Unfolded Protein Response (UPR^mt^) [44], we evaluated whether C/EBPδ silencing exacerbates the UPR^mt^. This was done by evaluating the expression of LONP1, ClpP and HSP60, well-known markers of the UPR^mt^ [44]. There were not, however, noticeable differences in the regulation of these three UPR^mt^ markers in cytokine-treated C-EBPδ-deficient INS-1E cells as compared to their control counterparts (Figure S4C), making it unlikely that the UPR^mt^ play a major role in the pro-apoptotic effect of C/EBPδ-silencing.

### The expression of the pro-apoptotic protein BIM is up-regulated in C/EBPδ-silenced cells

**Figure 4:**
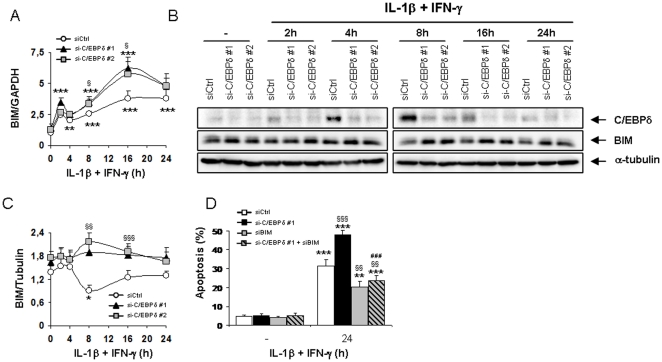
C/EBPδ-silencing increases the expression of the pro-apoptotic Bcl-2 family member BIM. (A–C) INS-1E cells were transfected with siCtrl (white dots), siC/EBPδ #1 (black triangles) or siC/EBPδ #2 (grey squares) and subsequently left untreated, or treated with IL-1β+IFN-γ for the indicated time points. (A) BIM mRNA expression was assayed by RT-PCR and normalized for the housekeeping gene GAPDH; (B) BIM, C/EBPδ and α-tubulin expressions were evaluated by Western blot. (C) Mean optical density measurements of BIM Western blots corrected for α-tubulin (representative figure in B). (D) INS-1E cells were transfected with siCtrl (white bars), or with either siC/EBPδ #1 (black bars), siBIM (grey bars) or siC/EBPδ #1+siBIM (hatched grey bars) and subsequently left untreated, or treated with IL-1β+IFN-γ for 24 h as indicated. Apoptosis was then assessed by HO/PI staining. Results are mean ± SEM of 4–6 experiments; *: *p*<0.05, **: *p*<0.01 and ***: *p*<0.001 vs untreated transfected with the same siRNA; §: *p*<0.05, §§: *p*<0.01 and §§§: *p*<0.001 vs siCtrl treated with cytokines at the same time point; ###: *p*<0.001 vs siC/EBPδ #1 treated with cytokines at the same time point; ANOVA followed by Student's *t* test with Bonferroni correction.

We next evaluated the effect of C/EBPδ knockdown on the mRNA expression of pro-apoptotic members of the Bcl-2 family associated with cytokine-induced β-cell apoptosis [23]–[25]. The expression of DP5, PUMA and BIM mRNAs were all induced by IL-1β+IFN-γ in INS-1E cells, reaching a peak after 16 h of cytokine treatment (Figure 4A & Figure S5A & S5B). Interestingly, the cytokine-induced up-regulation of BIM mRNA was exacerbated by C/EBPδ silencing (Figure 4A), while C/EBPδ deficiency did not affect the induction of DP5 and PUMA mRNAs following cytokine treatment (Figure S5A & S5B). Of note, the cytokine-induced up-regulation of the anti-apoptotic Bcl-2 family member B-cell lymphoma-extra large (Bcl-XL) was also unaffected by C/EBPδ knockdown (Figure S5C). BIM expression at the protein level was stable between 8 and 16 h in cytokine-treated C/EBPδ-silenced cells while it decreased in siCtrl-transfected cells at 8 h (Figure 4B & 4C). This decrease in BIM expression in siCtrl-transfected cells after 8 h of cytokine treatment is probably not due to an off-target effect of the siCtrl, since a similar decrease in BIM expression was observed in untransfected INS-1E cells after 8 h of IL-1β+IFN-γ exposure (Figures S5D et S5E). As a whole, the data described above suggest that BIM plays a key role in the sensitization of C/EBPδ-silenced cells to the pro-apoptotic effects of cytokines. To test this hypothesis, we performed viability assays after parallel knockdown of C/EBPδ and BIM. BIM inhibition partially prevented IL-1β+IFN-γ-induced apoptosis in INS-1E cells (Figure 4D), while the double inhibition of C/EBPδ and BIM abrogated the potentiating effects of C/EBPδ knockdown on cytokine-induced apoptosis (Figure 4D), suggesting that increased BIM activity contributes for the exacerbation of apoptosis in cytokine-treated C/EBPδ silenced cells.

### C/EBPδ inhibition increases cytokine-induced β-cell chemokine production through defective control of STAT1 transcriptional activity

**Figure 5:**
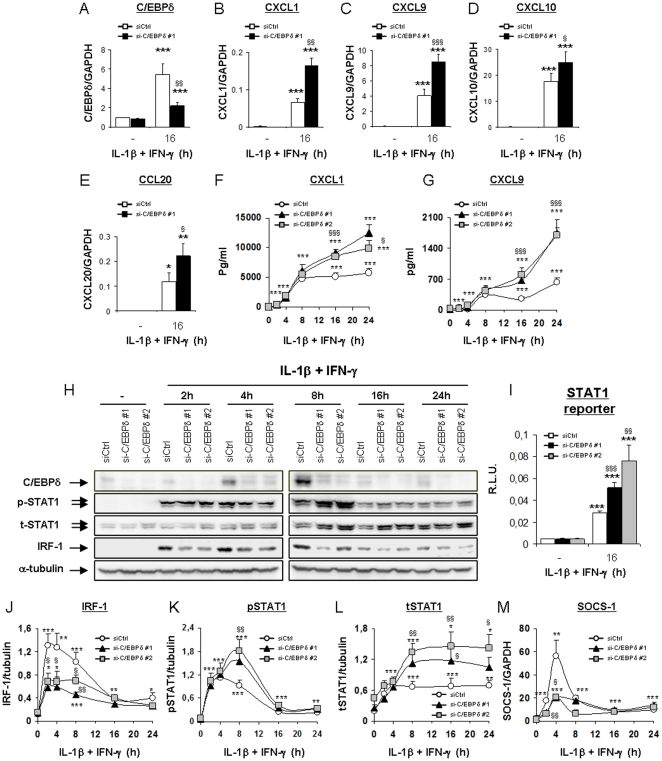
C/EBPδ silencing increases cytokine-induced chemokine production by enhancing STAT1 activation. INS-1E cells were transfected with either siCtrl (white dots/bars), siC/EBPδ #1 (black triangles/bars) or siC/EBPδ #2 (grey squares/bars) and subsequently left untreated, or treated with IL-1β+IFN-γ for the indicated time points; (A–E) C/EBPδ, CXCL1, 9, 10 & CCL20 mRNA expressions were assayed by RT-PCR and normalized for the housekeeping gene GAPDH; (F–G) CXCL1 and CXCL9 protein secretions were evaluated by ELISA. (H) C/EBPδ, phospho-STAT1, total STAT1, IRF-1 and α-tubulin expressions were evaluated by Western blot. (I) 24 h after siRNA transfection, cells were transfected with a STAT1 luciferase reporter+pRL-CMV and subsequently left untreated or exposed to cytokines for 16 h as indicated. Results are mean Relative Luciferase Unit (R.L.U.) ± SEM. (J–L) Mean optical density measurements of IRF1, phospho-STAT1 and total STAT1 Western blots corrected for α-tubulin (representative figure in H). (G) SOCS-1 mRNA expression was assayed by RT-PCR and normalized for the housekeeping gene GAPDH. Results are mean ± SEM of 4–6 experiments; *: *p*<0.05, **: *p*<0.01 and ***: *p*<0.001 vs untreated transfected with the same siRNA; §: *p*<0.05, §§: *p*<0.01 and §§§: *p*<0.001 vs siCtrl treated with cytokines at the same time point; ANOVA followed by Student's *t* test with Bonferroni correction.

β-cells produce many chemokines from the CXC and CC families in response to IL-1β+IFN-γ exposure, hence contributing to attract and activate immune cells during insulitis [1]. Taking this into account, we evaluated whether C/EBPδ also modulates the inflammatory response in IL-1β+IFN-γ-exposed cells. C/EBPδ silencing exacerbated the expression of CXCL1, 9, 10 and CCL20 mRNAs after 16 h of cytokine exposure (Figure 5A–E). These data were at least in part confirmed at the protein level, since C/EBPδ-silenced cells secreted higher amounts of the chemokines CXCL1 and CXCL9 as compared to control cells after 16- and 24 h of cytokine treatment (Figure 5F & 5G). Since we previously identified that the STAT1/IRF-1 signalling pathway exerts a key role in cytokine-induced chemokine production in β-cells [16], we performed time course analysis of the expression of these transcription factors in Ctrl- and C/EBPδ-deficient INS-1E cells after cytokine exposure. Interestingly, C/EBPδ silencing hampered cytokine-induced IRF-1 expression (Figure 5H & 5J). As previously shown [16], this impaired IRF-1 expression was accompanied by exacerbated STAT1 phosphorylation after 8 h of treatment with IL-1β+IFN-γ, but also by increased expression of total STAT1 after 8, 16 and 24 h of cytokine treatment (Figure 5H, 5K & 5L). This prolonged STAT1 activation in C/EBPδ-silenced cells enhanced STAT1 transcriptional activity, as shown by IL-1β+IFN-γ-induced augmented activation of a STAT1 reporter in C/EBPδ-silenced cells as compared to controls (Figure 5I). This effect is specific, since C/EBPδ knockdown had an opposite effect on an NF-κB reporter, inducing a 20–50% decrease (Figure S6). We next evaluated the expression of SOCS-1, a negative regulator of STAT1 activity that is modulated by IRF-1 activation in β-cells [16]. As shown in Figure 5M, cytokine-induced SOCS-1 up-regulation was also impaired in C/EBPδ-silenced cells as compared to Ctrl, reflecting the impaired activation of IRF-1 in C/EBPδ-deficient cells. All together, these results demonstrate that C/EBPδ exacerbates cytokine-induced chemokine production by interfering with IRF-1 up-regulation, hence disturbing the negative regulatory feedback loop by which IRF-1 modulates STAT1 activation through the induction of the inhibitory protein SOCS-1 [16].

### The transcription factors CHOP and STAT1 contribute to the enhancement of BIM expression in C/EBPδ-silenced cells

**Figure 6:**
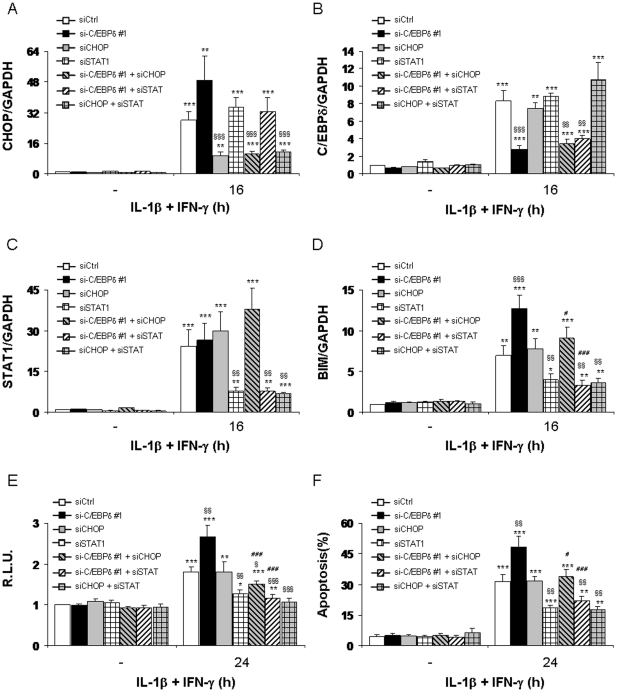
The transcription factors STAT1 and CHOP mediate cytokine-induced BIM up-regulation in C/EBPδ-silenced cells. INS-1E cells were transfected with siCtrl (white bars), siC/EBPδ #1 (black bars), siCHOP (grey bars), siSTAT1 (grid bars), siC/EBPδ #1+siCHOP (hatched grey bars), siC/EBPδ #1+siSTAT1 (hatched bars) or siCHOP+siSTAT1 (grid grey bars) and subsequently left untreated or treated with IL-1β+IFN-γ for 16 or 24 h as indicated. (A–D) CHOP, C/EBPδ, STAT1 & BIM mRNA expressions were assayed by RT-PCR and normalized for the housekeeping gene GAPDH. (E) 24 h after siRNA transfection, cells were transfected with a BIM promoter reporter+pRL-CMV and subsequently left untreated or exposed to cytokines as indicated. Results are mean Relative Luciferase Unit (R.L.U.) ± SEM. (F) Apoptosis was assessed by HO/PI staining. Results are mean ± SEM of 4–5 experiments; *: *p*<0.05, **: *p*<0.01 and ***: *p*<0.001 vs untreated transfected with the same siRNA; §: *p*<0.05, §§: *p*<0.01 and §§§: *p*<0.001 vs siCtrl treated with cytokines at the same time point; #: *p*<0.05 and ###: *p*<0.001 vs siC/EBPδ #1 treated with cytokines at the same time point; ANOVA followed by Student's *t* test with Bonferroni correction.

We described above that the activation of the transcription factors CHOP and STAT1 are increased or prolonged in C/EBPδ-silenced cells (Figure 3D–E and Figure 5H–L). Since both CHOP and STAT1 have been reported to mediate BIM transcriptional regulation in thymocytes and β-cells respectively [26], [45], we next evaluated a putative role of these transcription factors in the exacerbation of BIM expression in C/EBPδ-silenced β-cells. To this end, INS-1E cells were transfected with previously validated siRNAs targeting C/EBPδ (Figure 2A–B), CHOP (Figure 3G) and STAT1 [16], [46] alone or in combination, and the expression of BIM mRNA evaluated. As shown in Fig. 6A–C, the individual siRNAs inhibited their target gene without affecting the expression of the other non-targeted genes evaluated. As described in Figures 3 and 4, C/EBPδ silencing exacerbated CHOP and BIM up-regulation as compared to controls after 16 h of cytokine treatment (Figure 6A & 6D), while the knockdown of CHOP alone had no effect on cytokine-induced BIM expression (Figure 6D). Interestingly, the double knockdown of C/EBPδ and CHOP reversed to a large extent the exacerbation of BIM expression induced by C/EBPδ silencing alone (Figure 6D). STAT1 silencing, alone or in combination with C/EBPδ inhibition, potently inhibited IL-1β+IFN-γ-induced BIM up-regulation (Figure 6D). The double knockdown of CHOP and STAT1 did not further inhibit cytokine-induced BIM expression as compared to STAT1 silencing alone (Figure 6D). We next studied the regulation of the rat BIM promoter using a luciferase reporter construct containing the complete sequence of the this promoter (−2454/+2658) [47]. To this end, INS-1E cells were successively transfected with specific siRNAs as indicated and then with the rat BIM luciferase reporter. C/EBPδ silencing augmented the up-regulation of the BIM promoter induced by IL-1β+IFN-γ treatment as compared to siCtrl-transfected cells, while the concomitant knockdown of C/EBPδ and CHOP reversed the exacerbating effect of C/EBPδ silencing on the induction of the BIM promoter (Figure 6E). The siSTAT1, alone or associated with C/EBPδ or CHOP inhibition also prevented cytokine-induced up-regulation of the BIM promoter (Figure 6E). Viability assays corroborated these observations, with CHOP silencing inhibiting the exacerbating pro-apoptotic effects of C/EBPδ inhibition in cytokine-treated INS-1E cells, while STAT1 silencing both protected the cells against cytokine-induced apoptosis and counteracted the pro-apoptotic effects of C/EBPδ silencing (Figure 6F). Theses results identify STAT1 as a key mediator of IL-1β+IFN-γ-induced BIM up-regulation in β-cells, and suggest that the transcription factor CHOP mediates the “exacerbating effect” of C/EBPδ silencing on cytokine-induced BIM up-regulation.

### C/EBPδ overexpression decreases BIM expression and partially protects INS-1E cells against cytokine-induced apoptosis

**Figure 7:**
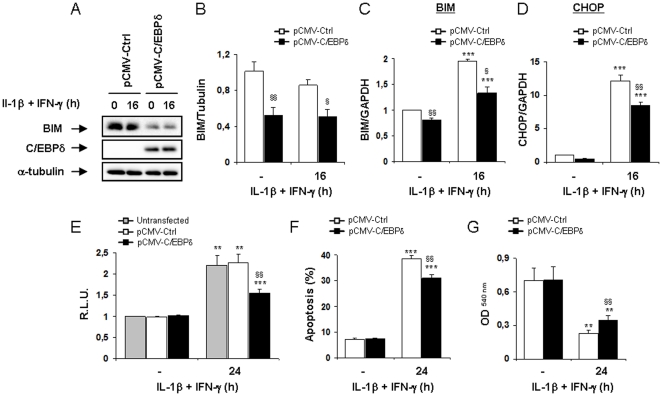
C/EBPδ overexpression decreases BIM expression and partially protect INS-1E cells against cytokine-induced apoptosis. INS-1E cells were left untransfected (grey bars) or transfected with pCMV-Ctrl (white bars) or pCMV-C/EBPδ (black bars) and subsequently left untreated or treated with IL-1β+IFN-γ for 16 or 24 h as indicated. (A) BIM, C/EBPδ and α-tubulin expressions were evaluated by Western blot. (B) Mean optical density measurements of BIM Western blots corrected for α-tubulin (representative figure in A). (C–D) BIM and CHOP mRNA expressions were assayed by RT-PCR and normalized for the housekeeping gene GAPDH. (E) Cells were co-tranfected with the vectors as decribed above and with a BIM promoter reporter+pRL-CMV and subsequently left untreated or exposed to cytokines as indicated. Results are mean Relative Luciferase Unit (R.L.U.) ± SEM; (F) Apoptosis was assessed by HO/PI staining; (G) Cell viability was evaluated using the neutral red-based toxicology kit. Results are mean ± SEM of 4–6 experiments; **: *p*<0.01 and ***: *p*<0.001 vs untreated untransfected or transfected with the same plasmid vector; §: *p*<0.05, §§: *p*<0.01 and §§§: *p*<0.001 vs pCMV-Ctrl treated with cytokines at the same time point; ANOVA followed by Student's *t* test with Bonferroni correction.

We next tested whether C/EBPδ overexpression affects BIM expression in INS-1E cells. Interestingly, a strong overexpression of C/EBPδ (Figure 7A) reduced both basal and cytokine-induced BIM expression (Figure 7A & 7B) and also decreased IL-1β+IFN-γ-induced up-regulation of BIM and CHOP mRNA expressions (Figure 7C & 7D). The co-tranfection of the C/EBPδ overexpression vector with the rat BIM promoter also reduced cytokine-induced up-regulation of this promoter as compared to untranfected- and control-transfected counterparts (Figure 7E). Finally, viability experiments demonstrated that C/EBPδ overexpression induced a moderate protection against IL-1β+IFN-γ-induced apoptosis (Figure 7F), and increased the survival of INS-1E cells as compared to control-transfected cells (Figure 7G). Taken together, these experiments support the hypothesis that C/EBPδ inhibits BIM expression and thus partially counteracts cytokine-induced apoptosis.

## Discussion

**Figure 8:**
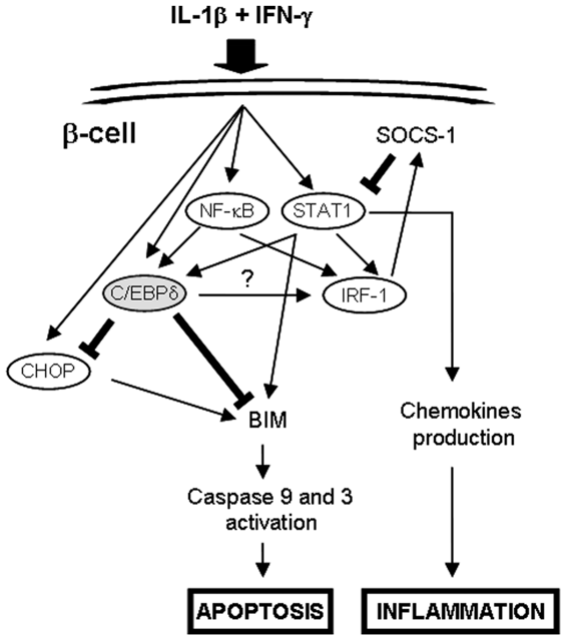
Suggested model for the role of C/EBPδ signalling in cytokine-treated β-cells. Transcription factors are highlighted in circle, thin arrows represents positive (inducing) effects, while bold T-shaped lines represent inhibitory effects. Upon IL-1β+IFN-γ exposure, C/EBPδ is up-regulated downstream of the transcription factors NF-κB and STAT1. Once synthesized, it inhibits CHOP and BIM expression, hampering cytokine-induced caspase 9 and 3 cleavage and apoptosis. C/EBPδ also exerts a positive role on cytokine-induced IRF-1 expression, which, via the induction of the regulator SOCS-1, down-regulates STAT1 activation and STAT1-driven chemokine production.

A better understanding of the signalling pathways involved in cytokine-induced β-cell apoptosis during insulitis may help to define potential therapeutic targets to interfere with T1D development [9]. We presently demonstrate, using silencing and overexpression approaches, that the transcription factor C/EBPδ is up-regulated by IL-1β+IFN-γ in rat β-cells and human islets and exerts regulatory functions in these cells, inhibiting pro-apoptotic and pro-inflammatory signals (Figure 8). NF-κB plays an important role in cytokine-induced C/EBPδ expression, as IL-1β (a known NF-κB inducer) up-regulates C/EBPδ expression in β-cells, while NF-κB blockade inhibits cytokine-induced C/EBPδ mRNA up-regulation (present data; [15]). These results are in line with previous observations describing NF-κB recruitment at the C/EBPδ promoter after 1 h of LPS exposure in macrophages [48]. The transcription factor STAT1 is also involved in cytokine-induced C/EBPδ expression, since IFN-γ enhances IL-1β-induced-C/EBPδ up-regulation and interfering with STAT1 activity hampers cytokine-induced C/EBPδ expression at early time points (Figure S1D; [16]). It is surprising, however, that the combination of TNF-α+IFN-γ does not up-regulate C/EBPδ expression in β-cells, and likewise, that C/EBPδ silencing or overexpression does not affect TNF-α+IFN-γ-induced cells death in β-cells (Figure S2B–C and data not shown). Indeed, TNF-α is also an inducer of NF-κB activation in these cells, albeit to a lesser extent than IL-1β [49]. C/EBPδ was shown to auto-regulate its expression by binding to sites located downstream of the C/EBPδ gene [43], [50]. Unlike IL-1β, TNF-α alone does not up-regulate C/EBPδ expression (Figure 1B–C). It is thus conceivable that C/EBPδ transcription in β-cells is initiated by NF-κB and other transcription factors induced by IL-1β (but not by TNF-α) and that this initial synthesis of C/EBPδ is required to allow further C/EBPδ-mediated auto-transcription. Interestingly, IL-1β+IFN-γ-induced C/EBPδ mRNA up-regulation was sustained until 24 h of cytokine treatment while the expression of C/EBPδ protein returned to basal after 16 h (Figs. 1 & 2). This may be explained by the progressive increase of cytokine-induced ER-stress, previously suggested to inhibit mRNA translation of many proteins in β-cells [42].

Inhibiting C/EBPδ activity in β-cells exacerbates IL-1β+IFN-γ-induced apoptosis by increasing the expression of the pro-apoptotic factors CHOP and BIM, while overexpressing C/EBPδ down-regulates CHOP and BIM expression and partially protects β-cells against the deleterious effects of cytokines (Figure 3, 4 & 7). This increased cytokine-induced apoptosis in C/EBPδ-silenced cells is independent of increased oxidative or nitrosative stress (Figure 2G & S2D–E), and our preliminary experiments did not indicate a role of modified autophagy in this process (data not shown). Autophagy has recently been suggested to be involved in β-cell death in both type 1 & type 2 diabetes [51], [52]. This negative regulatory role of C/EBPδ on cytokine-induced apoptosis in β-cells appears to be critical and non-redundant, since it is conserved between rodents and humans (Figures 1 and 2). This adds a new dimension to C/EBPδ functions, as previous reports described C/EBPδ mostly as a cell growth regulator and tumor suppressor. Indeed, C/EBPδ expression is down-regulated in acute myeloid leukemia [53] and in cervical, breast and liver cancers [54], [55], while C/EBPδ knockout mice display mammary gland ductal hyperplasia [37]. These anti-proliferative activities in mammary gland epithelial cells are linked to C/EBPδ-induced Cdc27 expression, resulting in proteasome-mediated destruction of the cell cycle promoting factor Cyclin D1 [38], [56]. Putative tumor-suppressing activities of C/EBPδ may, however, depend on the hormonal background, since C/EBPδ is highly expressed in androgen-dependent but not androgen-independent prostate carcinomas [57]. These data underline the context dependency of the function of regulatory transcription factors in different cell types or hormonal backgrounds, and indicate two novel functions for C/EBPδ in primary non-dividing cells namely as an important anti-apoptotic and anti-inflammatory regulator. Our preliminary data on C/EBPβ silencing suggest that C/EBPβ does not play a key role in IL-1β+IFN-γ-induced β-cell death. This differs from the previously reported effects of C/EBPβ in ER-stress-induced β-cell and tumoral cell death [41], [58], palmitate-induced β-cell apoptosis [59], and NO-dependent hepatocyte apoptosis [60]. On the other hand, C/EBPβ knockdown slightly decreases NO production in INS-1E cells (present data), which is in accordance with the reported roles of C/EBPα and C/EBPβ for iNOS induction in hepatocytes [60]. We cannot exclude that the moderate (60%) C/EBPβ silencing achieved in our experiments was not sufficient to disclose the putative effect of the transcription factor in cytokine-induced apoptosis. Additional experiments are required to clarify this issue.

Our data suggest that increased CHOP and BIM expression in C/EBPδ-silenced cells contributes for the exacerbation of apoptosis upon exposure to IL-1β+IFN-γ. Indeed, concomitant silencing of CHOP and C/EBPδ or BIM and C/EBPδ abrogates the exacerbating effects of C/EBPδ silencing on cytokine-induced up-regulation of the rat BIM promoter and on cytokine-induced apoptosis (present data). CHOP is a well known ER-stress-induced transcription factor that may trigger apoptosis through perturbation of intracellular pH and cellular cytoskeleton, down-regulation of the anti-apoptotic factor Bcl-2 and increase of reactive oxygen species production [61], [62]. CHOP expression is regulated by other C/EBP family members, including C/EBPα and C/EBPβ [63]. CHOP has been shown to hetero-dimerize with C/EBPα, β and δ [28], [43], [64], hence targeting these transcription factors to specific DNA binding sequences [65]. The nature of the interaction between C/EBPδ and CHOP in cytokine-treated β-cells and how C/EBPδ deficiency enhances CHOP expression, remain to be determined. Increased CHOP expression in C/EBPδ-silenced cells is associated with increased CHOP-regulated transcription, since the expression of the CHOP-regulated pro-apoptotic gene GADD34 [66] is also exacerbated in C/EBPδ-deficient cells (present data). Moreover, increased CHOP expression enhances the expression of the pro-apoptotic Bcl-2 family member BIM in cytokine-treated C/EBPδ-silenced cells (see below). Previous reports indicate that BIM has an important role in high glucose- [27], virus- [67] and cytokine-induced β-cell death [26], [68], which occurs through the mitochondrial apoptotic pathway [23]. We presently show that the exacerbation of cytokine-induced up-regulation of BIM mRNA and BIM promoter activity in C/EBPδ-silenced cells is at least in part mediated by the transcription factors STAT1 and CHOP. BIM up-regulation was already shown to be mediated by STAT1 in IL-21-treated lymphocytic leukaemia cells [69] and TNF-α+IFN-γ-exposed β-cells [26], while CHOP activity is required for BIM up-regulation and apoptosis induction in thapsigargin-treated thymocytes [45] and growth factor-deprived lymphoid precursors [70]. Although BIM mRNA was up-regulated by cytokines at all time points studied in siCtrl-tranfected cells, we observed a transient decrease in BIM protein expression after 8 h of IL-1β+IFN-γ exposure (Figure 4C). This is not due to an off-target effect of the siCtrl, as BIM protein also decreased after 8 h of cytokine exposure in untransfected cells (Figure S5D–E). This transient decrease in BIM protein expression was not observed in siC/EBPδ-silenced cells, in which exacerbated up-regulation of BIM mRNA expression seems to allow constant BIM protein expression throughout cytokine exposure. BIM has been reported to be targeted to proteasome-mediated degradation during rapid ischemic tolerance in neurons [71], [72] and subjected to caspase 3-mediated degradation as a feedback mechanism in MEFs and osteoclasts [73], but the putative pathways leading to BIM degradation and re-synthesis in IL-1β+IFN-γ-exposed β-cells remain to be clarified. As a whole, our data suggest that IL-1β+IFN-γ-induced BIM up-regulation in β-cells is mediated mostly via STAT1 activation, while CHOP seems to contribute for the exacerbating effects of C/EBPδ silencing on BIM expression. Nonetheless, while C/EBPδ overexpression repressed to a large extent BIM expression in INS-1E cells, it only moderately protected these cells against IL-1β+IFN-γ-induced apoptosis (Figure 7). These results emphasize the complexity of the pro-apoptotic pathways induced by cytokines in β-cells and the relative complementarity/redundancy of function of the pro-apoptotic Bcl-2 family members in cytokine-exposed β-cells [23].

Cytokine-induced up-regulations of the chemokines CXCL1, 9, 10 and CCL20 mRNAs are all exacerbated in C/EBPδ-silenced cells, as is the release of the tested chemokines CXCL1 and CXCL9, suggesting that C/EBPδ elicit anti-inflammatory activities in β-cells. These observations contrast with previous reports, describing C/EBPδ as a pro-inflammatory transcription factor that promotes LPS-induced activation of microglial cells and astrocytes [74], participates in TLR-induced pro-inflammatory cytokines production [39] and discriminates between transient and persistent LPS-mediated signalling in macrophages [48]. On the other hand, the anti-inflammatory agent dexamethasone was shown to induce C/EBPδ expression in skeletal muscle [57], while amyloid-β fibrils down-regulate C/EBPδ in astro-microglial cells during Alzheimer's disease [75]. We have previously shown that cytokine-induced chemokine production is increased by the prolongation of STAT1 activation in β-cells; IRF-1 controls a negative regulatory feedback loop that limits STAT1 activation through induction of the STAT1 regulator SOCS-1 [16]. In the present experiments, prolonged IL-1β+IFN-γ-induced STAT1 activation in C/EBPδ-silenced cells was also associated with defective induction of IRF-1 and its downstream gene SOCS-1, and augmented chemokine production. Of note, C/EBPδ deficiency decreases NF-κB-dependent transcription in cytokine-treated INS-1E cells. Since NF-κB is involved in cytokine-induced IRF-1 expression [16], [76] it is likely that C/EBPδ activation promotes IRF-1 expression by either participating directly to IRF-1 transcription by dimerizing with NF-κB1 p50 or RelA, or by inhibiting a negative regulator of NF-κB activation. The relation between C/EBPδ and IRF-1 expression remains to be clarified, and the co-immunoprecipitation experiments that we performed did not indicate a direct interaction between IRF-1 and C/EBPδ (data not shown), as it was previously reported for C/EBPβ in IFN-γ-treated HepG2 cells [77].

We and others observed that β-cells have in place regulatory mechanisms devoted to control excessive activation of deleterious factors induced by cytokines. These include PTPN2- and IRF-1/SOCS-1-mediated attenuation of STAT1 activation [16], [78], JunB-mediated attenuation of NO production and ER stress [79], A20- and SOCS-3-mediated NF-κB inactivation [80], [81] and HSP70 synthesis upon cytokine-treatment [82]. It is likely that C/EBPδ contributes to these signalling pathways that provide “local” regulation to inflammatory processes in order to protect poorly proliferating and long-lived cells, such as β-cells, against excessive damage. It remains to be clarified why these regulatory mechanisms are overruled in T1D, but the identification of novel agents that selectively promote protective pathways in β-cells may represent an alternative and “local” approach to protect β-cells against the relentless destructive assault initiated by autoimmune cells.

## Materials and Methods

### Culture of primary FACS-sorted rat β-cells, human islets and INS-1E cells

Male Wistar rats (Charles River Laboratories, Brussels, Belgium) were housed and used according to the guidelines of the Belgian Regulations for Animal Care; all experiments performed have been approved by the CEBEA Ethical Committee (Commission d'Ethique du Bien-Etre Animal, Universite Libre de Bruxelles, Permit Number LA 1230351, valid until 2014). For islet isolation, pancreases were digested by collagenase and islets were hand picked under a stereomicroscope. B-cells were purified by autofluorescence-activated cell sorting (FACSAria, BD Bioscience, San Jose, CA, USA) [83]. The preparations contained 95±2% β-cells (*n* = 9). β-cells were cultured for 2 days in Ham's F-10 medium containing 10 mM glucose, 2 mM glutaMAX, 50 µM 3-isobutyl-1-methylxanthine, 5% FBS, 0.5% charcoal-absorbed BSA (Boehringer, Indianapolis, IN, USA), 50 U/ml penicillin and 50 µg/ml streptomycin [83]. During cytokine exposure, cells were cultured in the same medium but without serum.

Human islets were isolated from 10 non-diabetic organ donors (age 65±4 years; body mass index 26.1±1 kg/m^2^) in Pisa, Italy, with the approval of the Ethics Committee of the University of Pisa. A written consent was obtained from each organ donor for the use of the pancreas for research purposes. Islets were isolated by enzymatic digestion, density-gradient purification [84], and cultured in M199 medium containing 5.5 mM glucose. The human islets were shipped to Brussels within 1–5 days of isolation. After overnight recovery in Ham's F-10 containing 6.1 mM glucose, 10% FBS, 2 mM GlutaMAX, 50 µM 3-isobutyl-1-methylxanthine, 1% BSA, 50 U/ml penicillin and 50 µg/ml streptomycin, islets were dispersed for viability assays or exposed to cytokines in the same medium without FBS for 24 h. The percentage of β-cells, examined in the 10 dispersed islet preparations by staining with anti-insulin antibody (1∶1000, Sigma, Bornem, Belgium) and donkey anti-mouse IgG rhodamine (1∶200, Lucron Bioproducts, De Pinte, Belgium), was 63±5%; only preparations containing >40% β-cells were used in the experiments.

The rat insulin-producing INS-1E cell line (a kind gift from Dr. C. Wollheim, Centre Medical Universitaire, Geneva, Switzerland) was cultured as previously described [85] and used between passages 52 and 72.

### RNA interference

The following: siRNAs were used (sequence 5′-3′): BLOCK-iT Stealth™ Select siRNA (Invitrogen, Paisley, UK) for rat siC/EBPδ #2 CCGACCUCUUCAACAGCAAUCACAA, human siC/EBPδ #1 ACAGCCUGGACUUACCACCACUAAA, human siC/EBPδ #2 GCCUCCGGCAGUUCUUCAAGCAGCU, human siC/EBPδ #3 CGAGAGAAGCUAAACGUGUUUAUUU, rat siSTAT1 CCCUAGAAGACUUACAAGAUGAAUA, rat siBIM CGAGGAGGGCGUUUGCAAACGAUUA; ON-TARGETplus SMARTpool® (Thermo Scientific, Chicago, IL, USA) for rat siC/EBPδ #1 Seq. #1 CGCAGACAGUGGUGAGCUU Seq. #2 CGACUUCAGCGCCUACAUU Seq. #3 GAUCUUCGCCGACCUCUUC Seq. #4 CCACGACCCCUGCCAUGUA and rat siCHOP Seq. #1 GGAAGAACUAGGAAACGGA Seq. #2 GGGCUCUGAUCGACCGCAU Seq. #3 CUGAAGAGAACGAGCGGCU Seq. #4 ACGAGGAAAUCGAGCGCCU; Silencer Select Pre-designed siRNA for rat siC/EBPβ #1 CUAUUUCUAUGAGAAAAGAtt, rat siC/EBPβ #2 GCAUUAAAGUGAAGACAUUtt. Allstars Negative Control siRNA was used for control-transfected conditions (Qiagen, Venlo, The Netherlands, sequence not provided). The concentration of siRNA used for cell transfection (30 nM) was selected based on previous dose-response studies [78], [86]; neither transfection nor the control siRNA affect β-cell viability, function or gene expression as compared to non-transfected β-cells [16], [17], [78]. In co-transfection experiments 30 nM of each active siRNA was used while 60 nM siCtrl siRNA was transfected in the control condition. Lipofectamine RNAiMAX lipid reagent (Invitrogen) was used for siRNA transfection, following the previously described protocol [78]. After transfection, cells were cultured for a 48 h recovery period and subsequently exposed to cytokines.

### Cell treatment and NO measurement

The following cytokine concentrations were used, based on previous dose-response experiments [8], [85], [87]: recombinant human IL-1β (specific activity 1.8×10^7^ units/mg; a gift from C.W. Reinolds, National Cancer Institute, Bethesda, MD) at 10 units/ml (for rat cells) or 50 units/ml (for human cells); recombinant murine TNF-α (specific activity: 2×10^8^ units/mg; Innogenetics, Gent, Belgium) at 1,000 units/ml; and recombinant rat or human IFN-γ (specific activity: 2×10^7^ units/mg; R&D Systems, Abingdon, U.K.) at 100 and 1,000 units/ml for rat cells and human islets respectively. Culture supernatants were collected for nitrite determination (nitrite is a stable product of NO oxidation) at OD^540 nm^ using the Griess method.

### Evaluation of intracellular ROS/RNS content

Cells were transfected with siCtrl, siC/EBPδ #1 or siC/EBPδ #2 and left untreated or treated as described above for 2–24 h with IL-1β+IFN-γ. Cells were then lysed in PBS by freeze/thaw cycles followed by sonication. Cell lysates were centrifuged to discard cellular debris and lysates were quantified using the Bradfod method. Intracellular ROS/RNS content was then evaluated in an equivalent amount of cell extract for each condition using the OxiSelect *in vitro* ROS/RNS assay kit (Green Fluorescence - Cell Biolabs, San Diego, CA, USA).

### Western blots

Cells were washed, lysed, extracts were resolved by 10–14% SDS-PAGE and transferred to a nitrocellulose membrane as described [78]. The antibodies used were: anti-C/EBPδ (sc-636), anti-STAT1 (sc-346), anti-CHOP (GADD153 – sc-575) and anti-IRF-1 (sc-640) from Santa Cruz Biotechnology (Santa Cruz, CA, USA); anti-phospho-STAT1 (Y701 - #9171), anti-BIM (#2819), anti-HSP60 (#4870), anti-cleaved caspase 3 (#9661) and anti-cleaved caspase 9 (#9507) from Cell Signalling (Danvers, MA, USA); anti ClpP (15698-1-AP) and anti-LONP1 (15440-1-AP) from Proteintech (Manchester; UK); anti-α-tubulin (T9026) from Sigma (Bornem, Belgium). HRP-conjugated anti-rabbit or anti-mouse IgG (Lucron Bioproducts, De Pinte, Belgium) were used as secondary antibodies. Immunoreactive bands were revealed using the SuperSignal West Femto chemiluminescent substrate (Thermo Scientific), detected using a LAS-3000 CCD camera and quantified with the Aida Analysis software (Fujifilm).

### mRNA extraction and real time PCR

Poly(A)^+^ mRNA was isolated from INS-1E cells or rat primary β-cells using the Dynabeads mRNA DIRECT™ kit (Invitrogen), and reverse transcribed as previously described [83]. The real time PCR amplification reaction was done as described [83], using SYBR Green and compared to a standard curve. Expression values were corrected for the housekeeping genes glyceraldehyde-3-phosphate dehydrogenase (GAPDH) and β-actin for rat and human assays respectively; we have previously shown that cytokines do not modify GAPDH and β-actin expressions in these species [78]. The primers used in this study are listed in Table S1.

### Assessment of apoptosis and viability

The percentage of viable, apoptotic and necrotic cells was determined after staining with the DNA-binding dyes Propidium Iodide (PI, 5 µg/ml, Sigma) and Hoechst 33342 (HO, 5 µg/ml, Sigma) [83]. This method is quantitative, and has been validated by systematic comparison against electron microscopy [88] and several other well-characterized methods, including fluorometric caspase 3 & 7 assays and determination of histone-complexed DNA fragments by ELISA [24], [25], [89]–[91]. A minimum of 500 cells was counted in each experimental condition. Viability was evaluated by two independent observers, one of them being unaware of sample identity. The agreement between findings obtained by the two observers was >90%. Results are expressed as percent apoptosis, calculated as (number of apoptotic cells/total number of cells)×100. Apoptosis was confirmed in some experiments by the Cell Death Detection ELISAplus kit (Roche Diagnostics, Vilvoorde, Belgium), which detects cytoplasmic fragmented DNA. For some key experiments, cellular viability after cytokine treatment was assessed using the Neutral Red-based *in vitro* toxicology assay kit (Sigma) as follows: neutral red (final concentration 33‰) was added to the culture medium for 2 h before the end of cytokine treatment. The culture medium was then removed and cells were washed once with the fixative solution to discard detached cells and remove unincorporated neutral red. The neutral red incorporated into living cells was then eluted using the solubilisation solution. ODs were read at 540 nm; the magnitude of the OD^540 nm^ is directly proportional to the number of remaining living cells for each condition.

### Luciferase reporter assay and immunofluorescence

INS-1E cells were transfected as previously described [49] with pRL-CMV encoding Renilla luciferase (Promega) and either a luciferase promoter-reporter construct containing 3 GAS consensus sequences (STAT1 reporter) or 5 NF-κB consensus binding sites (NF-κB reporter) or the fragment (−2545/+2658) from the rat BIM promoter (BIM promoter experiments – a kind gift of Pr. M. Li, Sun Yat-sen University, China) [47]. Luciferase activities were assayed after 16–24 h of cytokine treatment [49].

For immunofluorescence studies, INS-1E cells were seeded onto polylysine-coated glass coverslips and treated as indicated. Cells were washed with cold PBS, fixed for 10 min in 4% paraformeldehyde and permeabilized for 5 min in PBS containing 0.1% Triton X-100 (PBST). Following a 1 h blocking with 5% normal goat serum (NGS) in PBST, cells were incubated overnight with the following primary antibodies diluted at 1/1000 in PBST 5% NGS: anti-Bax (sc-493, Santa Cruz Biotechnology), anti-AIF (#4642, Cell Signaling), anti-ATP synthase β (A9728, Sigma) or mouse anti-cytochrome *c* (# 556432, BD Pharmingen, San Jose, CA, USA). Cells were washed three times with PBS and DyLight^488^-conjugated donkey anti-mouse and DyLight^549^-conjugated donkey anti-rabbit antibodies (Lucron Bioproducts) were applied for 2 h at 1∶1000 in PBST. After 2 washes with PBST, nuclei were counterstained with Hoechst 33342 (HO) for 5 min, and washed 3 times with PBS. Coverslips were mounted in 50% glycerol, and immunofluorescence was visualized on a Zeiss Axiovert 200 microscope (Oberkochen, Germany).

### Glucose oxidation assay

D-[U-^14^C] glucose (specific activity: 300 mCi/mmol, concentration: 1 mCi/ml, Perkin Elmer, Waltham, MA) was used to evaluate glucose oxidation in untreated and IL-1β+IFN-γ-treated siCtrl- or siC/EBPδ-transfected cells. Cells were trypsinized and resuspended in KRBH solution without glucose at 5×10^6^ living cells/ml. 20 µl of the cell suspension (1.10^5^ cells) was transferred to glass vials containing 20 ml KRBH buffer supplemented with 0.19 mCi of D-[U-^14^C] glucose and non-radioactive glucose to a final concentration of 1.67, 10 or 16.7 mM of glucose. The vials were inserted into 20 ml glass scintillation flasks, gassed with 95% CO_2_ and 5% O_2_, airtight sealed with rubber membranes and shaken continuously for 2 h at 37°C. After incubation, 0.02 ml of metabolic poison (400 mM citrate buffer - pH 4.9, 3 mM KCN, 10 mM Antimycin A, 20 mM Rotenone; pH 7.4) was injected through the rubber cap into the vials containing the cells and 0.2 ml hyamine hydroxide was injected outside of the vials to absorb the released CO_2_. Following 1 h incubation at 37°C, the vials were removed and scintillation fluid (6 ml) was added to the hyamine. After 14 h at 4°C, the radioactivity was measured in a TriCarb 2100TR Liquid scintillation analyzer (Perkin Elmer, Waltham, MA). The rate of glucose oxidation was expressed as µmol/120 min.1000 cells.

### Infection with recombinant adenoviruses

Cells were infected either with Ad-Luc (luciferase-expressing adenovirus) or Ad-srIκB (a virus expressing an NF-κB super-repressor IκBα protein) [40]. Cells were infected for 2 h at 37°C with a multiplicity of infection (MOI) of 10. The MOI was selected based on lowest toxicity by viral infection combined with highest blockade of NF-κB activation. After infection (24 h), cells were treated with cytokines. We have previously shown that infection of β-cells with Ad-srIκB at the MOI used in the present study does not change its function or viability [40].

### Overexpression of rat C/EBPδ

The expression vectors pCMV-Ctrl [16] and pCMV-C/EBPδ (TrueORF cDNA Clones - OriGene, Rockville, MD, USA) were transfected in INS-1E cells using Lipofectamine 2000 (Invitrogen) as previously described [49]. After overnight incubation, the medium was changed and cells were exposed to cytokines as indicated.

### Evaluation of chemokine accumulation in the medium by ELISAs

Cells were transfected with siCtrl, siC/EBPδ #1 or siC/EBPδ #2 and subsequently left untreated or treated with IL-1β+IFN-γ as described above. Supernatants were collected after 2, 4, 8, 16 & 24 h of treatment for determination of CXCL1 and CXCL9 chemokines secretion using the commercially available ELISA kits for rat CXCL1 (R&D Systems) and rat CXCL9 (Uscn Life Science, Wuhan, China).

### Statistical analysis

Data are presented as mean ± SEM. Comparisons were performed by two-tailed paired Student's *t*-test or by ANOVA followed by Student's *t* test with Bonferroni correction as indicated. A *p* value<0.05 was considered statistically significant.

## Supporting Information

Figure S1 **IL-1β and IFN-γ up-regulate C/EBPδ expression in INS-1E cells and primary FACS-sorted rat β-cells.** (A) INS-1E cells were left untreated or treated for 8 h with either IL-1β, IFN-γ, TNF-α, IL-1β+IFN-γ or TNF-α+IFN-γ. The figure represents mean optical density measurements of C/EBPδ Western blots corrected for α-tubulin (representative figure in Figure 1C). (B) Primary purified rat β-cells were left untreated or treated with the combination of TNF-α+IFN-γ for 24 h; C/EBPδ mRNA expression was assayed by RT-PCR and normalized for the housekeeping gene GAPDH. (C) INS-1E cells were infected with Ad-Luc or Ad-srIκB and subsequently treated with cytokines as indicated. (D) INS-1E cells were transfected with siCtrl (white dots) or siSTAT1 (black triangles) and subsequently left untreated, or treated with IL-1β+IFN-γ for 12- or 24 h as indicated. (C–D) C/EBPδ mRNA expression were assayed by RT-PCR and normalized for the housekeeping gene GAPDH. Results are mean ± SEM of 4–5 independent experiments; *: *p*<0.05, **: *p*<0.01 and ***: *p*<0.001 vs untreated (transfected with the same siRNA); §: *p*<0.05 and §§: *p*<0.01 vs siCtrl treated with cytokines at the same time point; ANOVA followed by Student's *t* test with Bonferroni correction.
(TIF)

Figure S2 **C/EBPδ-silencing neither exacerbates IL-1β- or IFN-γ-induced apoptosis nor aggravates cytokine-induced inhibition of glucose oxidation in INS-1E cells.** (A–E) INS-1E cells were transfected with either siCtrl (white dots/bars), siC/EBPδ #1 (black triangles/bars) or siC/EBPδ #2 (grey squares/bars) and subsequently left untreated, or treated with either IL-1β, IFN-γ, TNF-α, IL-1β+IFN-γ or TNF-α+IFN-γ for the indicated time point. (A–B) Apoptosis was assessed by HO/PI staining after 24 h; (C) Cell viability was evaluated using the neutral red-based toxicology kit. (D) Nitrite production was evaluated as described in Materials & Methods; (E) ROS and RNS content was evaluated as described in Materials & Methods. (F) HeLa cells were transfected with siCtrl or 3 siRNAs targeting human C/EBPδ (#1, #2 & #3) and subsequently left untreated or treated with IL-1β+IFN-γ for 8 h. C/EBPδ and α-tubulin expressions were evaluated by Western blot. (G–H) INS-1E cells were transfected with rat siC/EBPδ #1 and subsequently treated with IL-1β+IFN-γ for 16 h. Cells were fixed and processed for immunofluorescence as described in Methods; A: apoptotic cell, L: living cell. The figure is representative of 5 independent experiments. (I–J) Mean optical density measurements of cleaved caspase 3 & 9 Western blots corrected for α-tubulin (representative figure in Figure 2I). (K) Glucose oxidation assay was performed as described in Materials & Methods. Results are mean ± SEM of 4–5 independent experiments; *: *p*<0.05, **: *p*<0.01 and ***: *p*<0.001 vs untreated transfected with the same siRNA; §: *p*<0.05, §§: *p*<0.01 and §§§: *p*<0.001 vs siCtrl treated with cytokines at the same time point; ANOVA followed by Student's *t* test with Bonferroni correction.
(TIF)

Figure S3 **C/EBPβ silencing doesn't affect IL-1β+IFN-γ-induced apoptosis and nitrite production in INS-1E cells.** (A) INS-1E cells were left untreated or treated with the combination of IL-1β+IFN-γ for 12 or 24 h as indicated and C/EBPβ mRNA expression was assayed by RT-PCR and normalized for the housekeeping gene GAPDH. (B–D) INS-1E cells were transfected with siCtrl (white bars), siC/EBPβ #1 (black bars) or siC/EBPβ #2 (grey bars) and subsequently left untreated, or treated with IL-1β+IFN-γ for 24 h as indicated. (B) C/EBPβ mRNA expression was assayed by RT-PCR and normalized for the housekeeping gene GAPDH. (C) Apoptosis was assessed by HO/PI staining. (D) Nitrite production was evaluated as described in Materials & Methods. Results are mean ± SEM of 4 independent experiments; *: *p*<0.05, **: *p*<0.01 and ***: *p*<0.001 vs untreated transfected with the same siRNA; §: *p*<0.05 and §§: *p*<0.01 vs siCtrl treated with cytokines at the same time point; ANOVA followed by Student's *t* test with Bonferroni correction.
(TIF)

Figure S4 **Expressions of XBP-1s, BIP and the UPR^mt^ markers are unaffected by C/EBPδ silencing in INS-1E cells.** (A–C) INS-1E cells were transfected with siCtrl (white dots), siC/EBPδ #1 (black triangles) or siC/EBPδ #2 (grey squares) and subsequently left untreated, or treated with IL-1β+IFN-γ for the indicated time points. (A–B) XBP-1s and BIP mRNA expression were assayed by RT-PCR and normalized for the housekeeping gene GAPDH. (C) The UPR^mt^ markers LONP1, HSP60, ClpP and α-tubulin expressions were evaluated by Western blot; the figure shown is representative of 3 independent experiments. Results in Figs. 4A & 4B are mean ± SEM of 4 independent experiments; **: *p*<0.01 and ***: *p*<0.001 vs untreated transfected with the same siRNA; ANOVA followed by Student's *t* test with Bonferroni correction.
(TIF)

Figure S5 **C/EBPδ knockdown doesn't affect cytokine-induced up-regulation of DP5, PUMA and Bcl-XL in INS-1E cells.** (A–C) INS-1E cells were transfected with siCtrl (white dots), siC/EBPδ #1 (black triangles) or siC/EBPδ #2 (grey squares) and subsequently left untreated or treated with IL-1β+IFN-γ for the indicated time points. DP5, PUMA and Bcl-XL expressions were assayed by RT-PCR and normalized for the housekeeping gene GAPDH. (D–E) INS-1E cells were left untransfected (grey dots) or transfected with siCtrl (white dots) and subsequently left untreated or treated with IL-1β+IFN-γ for the indicated time points. (D) BIM and α-tubulin expression were evaluated by Western blot. (E) Mean optical density measurements of BIM Western blots corrected for α-tubulin (representative figure in E). Results are mean ± SEM of 4–5 independent experiments; *: *p*<0.05, **: *p*<0.01 and ***: *p*<0.001 vs untreated untransfected or transfected with the same siRNA; ANOVA followed by Student's *t* test with Bonferroni correction.
(TIF)

Figure S6 **C/EBPδ silencing decreases the activation of an NF-κB luciferase reporter.** INS-1E cells were transfected with siCtrl (white bars), siC/EBPδ #1 (black bars) or siC/EBPδ #2 (grey bars). After 24 h, cells were transfected with a NF-κB luciferase reporter+pRL-CMV and subsequently left untreated or exposed to IL-1β+IFN-γ for 16 h as indicated. Results are mean Relative Luciferase Unit (R.L.U.) ± SEM of 5 independent experiments; ***: *p*<0.001 vs untreated transfected with the same siRNA; §§: *p*<0.01 and §§§: *p*<0.001 vs siCtrl treated with cytokines at the same time point; ANOVA followed by Student's *t* test with Bonferroni correction.
(TIF)

Table S1 **List of primers used for real time PCR.**
(TIF)
