# Supplementary figures and images for: The Transcription Factor C/EBP delta Has Anti-Apoptotic and Anti-Inflammatory Roles in Pancreatic Beta Cells
Source: PLoS One. 2012 Feb 8;7(2):e31062. doi: 10.1371/journal.pone.0031062 (PMC3275575; doi:10.1371/journal.pone.0031062)

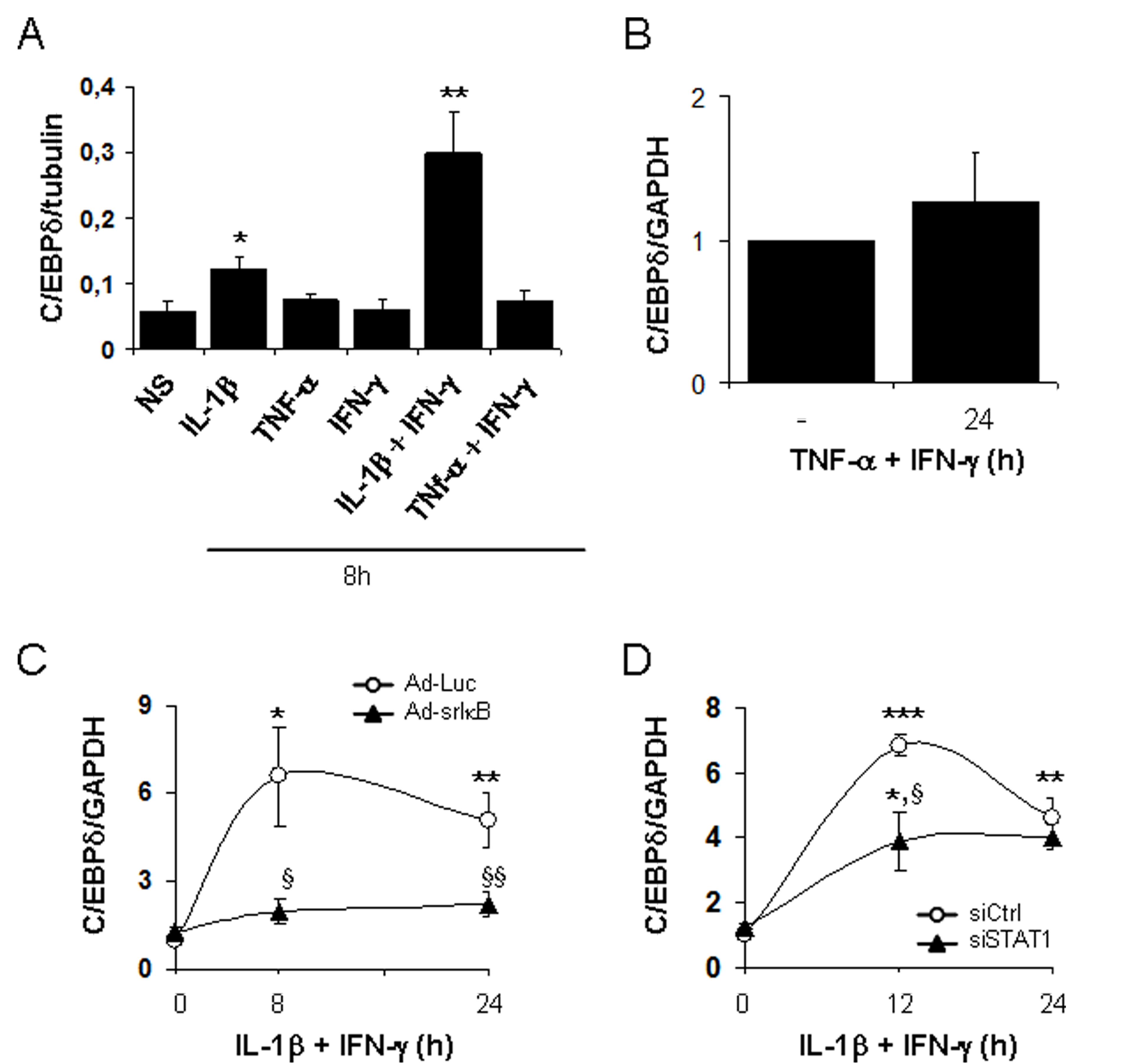

Supplement: Figure S1 — IL-1β and IFN-γ up-regulate C/EBPδ expression in INS-1E cells and primary FACS-sorted rat β-cells. (A) INS-1E cells were left untreated or treated for 8 h with either IL-1β, IFN-γ, TNF-α, IL-1β+IFN-γ or TNF-α+IFN-γ. The figure represents mean optical density measurements of C/EBPδ Western blots corrected for α-tubulin (representative figure in Figure 1C). (B) Primary purified rat β-cells were left untreated or treated with the combination of TNF-α+IFN-γ for 24 h; C/EBPδ mRNA expression was assayed by RT-PCR and normalized for the housekeeping gene GAPDH. (C) INS-1E cells were infected with Ad-Luc or Ad-srIκB and subsequently treated with cytokines as indicated. (D) INS-1E cells were transfected with siCtrl (white dots) or siSTAT1 (black triangles) and subsequently left untreated, or treated with IL-1β+IFN-γ for 12- or 24 h as indicated. (C–D) C/EBPδ mRNA expression were assayed by RT-PCR and normalized for the housekeeping gene GAPDH. Results are mean ± SEM of 4–5 independent experiments; *: p<0.05, **: p<0.01 and ***: p<0.001 vs untreated (transfected with the same siRNA); §: p<0.05 and §§: p<0.01 vs siCtrl treated with cytokines at the same time point; ANOVA followed by Student's t test with Bonferroni correction. (TIF) [file pone.0031062.s001.tif]

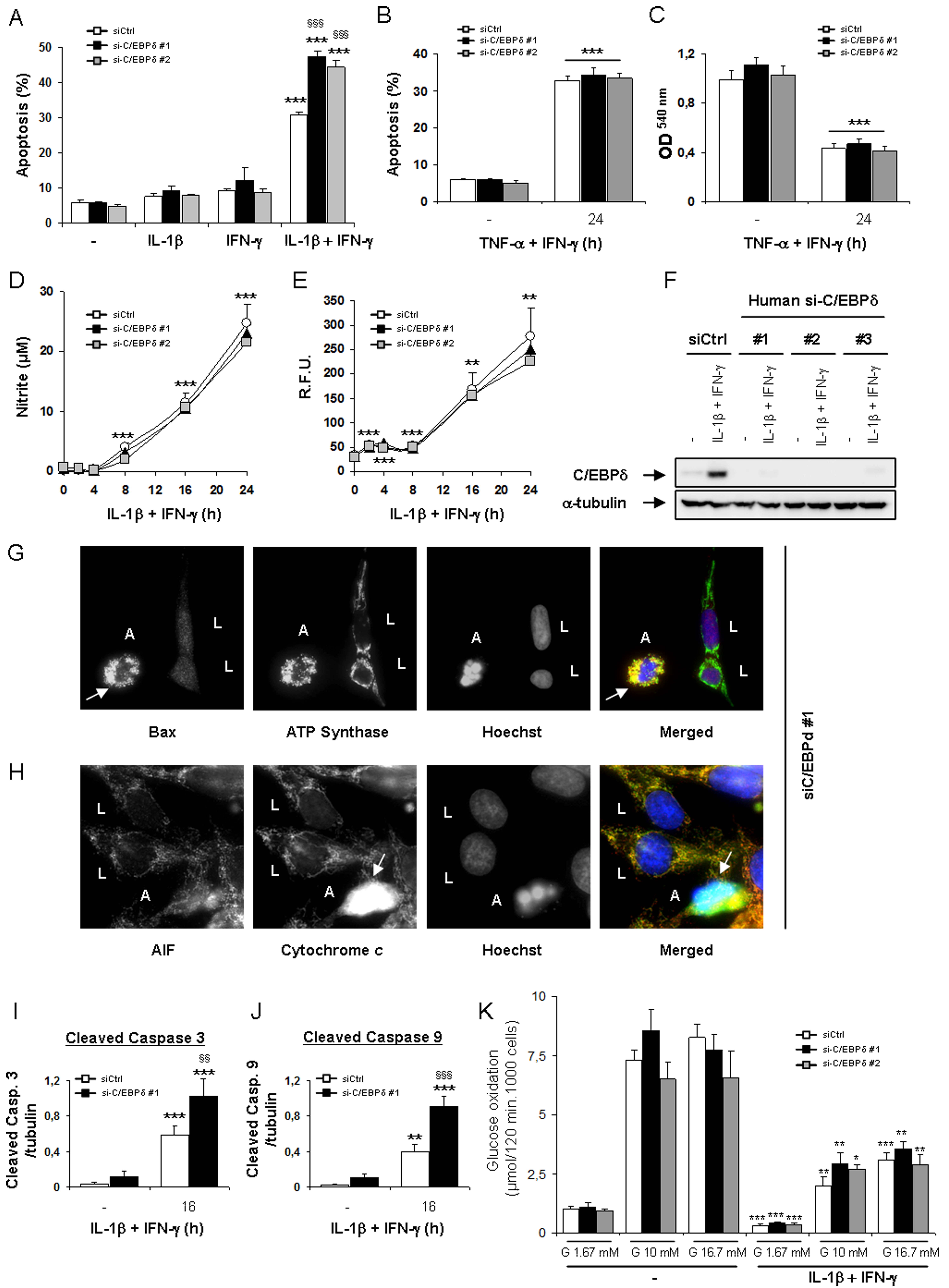

Supplement: Figure S2 — C/EBPδ-silencing neither exacerbates IL-1β- or IFN-γ-induced apoptosis nor aggravates cytokine-induced inhibition of glucose oxidation in INS-1E cells. (A–E) INS-1E cells were transfected with either siCtrl (white dots/bars), siC/EBPδ #1 (black triangles/bars) or siC/EBPδ #2 (grey squares/bars) and subsequently left untreated, or treated with either IL-1β, IFN-γ, TNF-α, IL-1β+IFN-γ or TNF-α+IFN-γ for the indicated time point. (A–B) Apoptosis was assessed by HO/PI staining after 24 h; (C) Cell viability was evaluated using the neutral red-based toxicology kit. (D) Nitrite production was evaluated as described in Materials & Methods; (E) ROS and RNS content was evaluated as described in Materials & Methods. (F) HeLa cells were transfected with siCtrl or 3 siRNAs targeting human C/EBPδ (#1, #2 & #3) and subsequently left untreated or treated with IL-1β+IFN-γ for 8 h. C/EBPδ and α-tubulin expressions were evaluated by Western blot. (G–H) INS-1E cells were transfected with rat siC/EBPδ #1 and subsequently treated with IL-1β+IFN-γ for 16 h. Cells were fixed and processed for immunofluorescence as described in Methods; A: apoptotic cell, L: living cell. The figure is representative of 5 independent experiments. (I–J) Mean optical density measurements of cleaved caspase 3 & 9 Western blots corrected for α-tubulin (representative figure in Figure 2I). (K) Glucose oxidation assay was performed as described in Materials & Methods. Results are mean ± SEM of 4–5 independent experiments; *: p<0.05, **: p<0.01 and ***: p<0.001 vs untreated transfected with the same siRNA; §: p<0.05, §§: p<0.01 and §§§: p<0.001 vs siCtrl treated with cytokines at the same time point; ANOVA followed by Student's t test with Bonferroni correction. (TIF) [file pone.0031062.s002.tif]

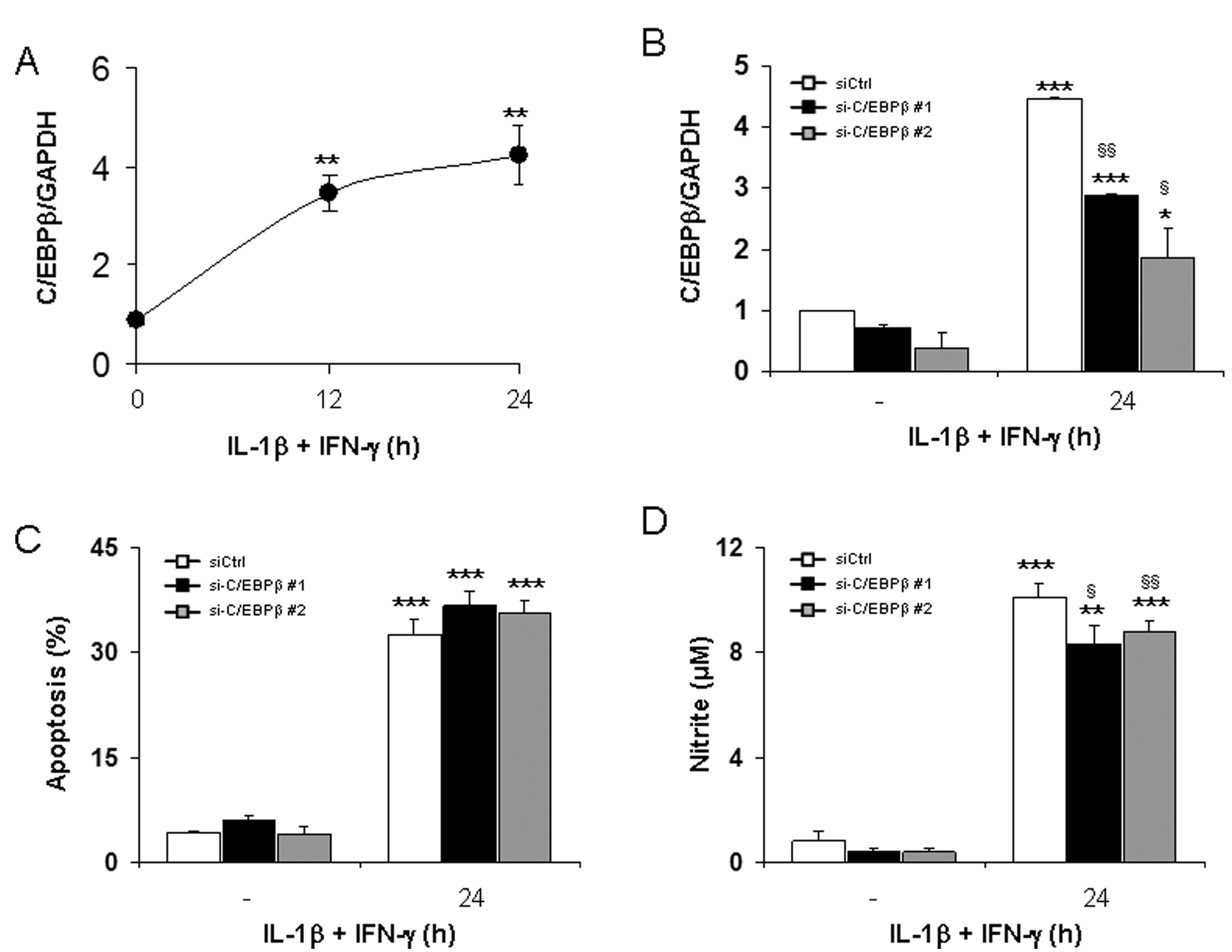

Supplement: Figure S3 — C/EBPβ silencing doesn't affect IL-1β+IFN-γ-induced apoptosis and nitrite production in INS-1E cells. (A) INS-1E cells were left untreated or treated with the combination of IL-1β+IFN-γ for 12 or 24 h as indicated and C/EBPβ mRNA expression was assayed by RT-PCR and normalized for the housekeeping gene GAPDH. (B–D) INS-1E cells were transfected with siCtrl (white bars), siC/EBPβ #1 (black bars) or siC/EBPβ #2 (grey bars) and subsequently left untreated, or treated with IL-1β+IFN-γ for 24 h as indicated. (B) C/EBPβ mRNA expression was assayed by RT-PCR and normalized for the housekeeping gene GAPDH. (C) Apoptosis was assessed by HO/PI staining. (D) Nitrite production was evaluated as described in Materials & Methods. Results are mean ± SEM of 4 independent experiments; *: p<0.05, **: p<0.01 and ***: p<0.001 vs untreated transfected with the same siRNA; §: p<0.05 and §§: p<0.01 vs siCtrl treated with cytokines at the same time point; ANOVA followed by Student's t test with Bonferroni correction. (TIF) [file pone.0031062.s003.tif]

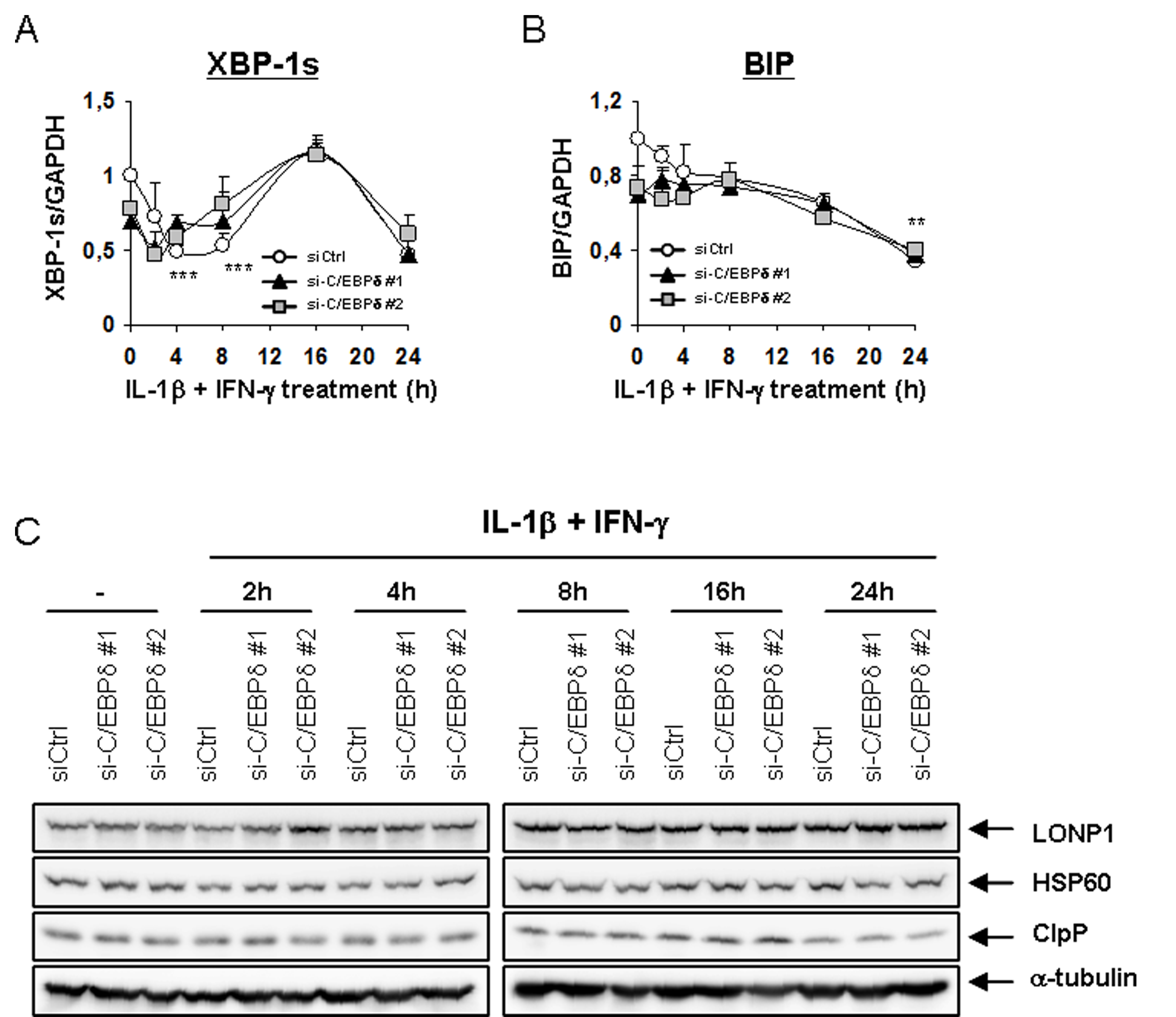

Supplement: Figure S4 — Expressions of XBP-1s, BIP and the UPRmt markers are unaffected by C/EBPδ silencing in INS-1E cells. (A–C) INS-1E cells were transfected with siCtrl (white dots), siC/EBPδ #1 (black triangles) or siC/EBPδ #2 (grey squares) and subsequently left untreated, or treated with IL-1β+IFN-γ for the indicated time points. (A–B) XBP-1s and BIP mRNA expression were assayed by RT-PCR and normalized for the housekeeping gene GAPDH. (C) The UPRmt markers LONP1, HSP60, ClpP and α-tubulin expressions were evaluated by Western blot; the figure shown is representative of 3 independent experiments. Results in Figs. 4A & 4B are mean ± SEM of 4 independent experiments; **: p<0.01 and ***: p<0.001 vs untreated transfected with the same siRNA; ANOVA followed by Student's t test with Bonferroni correction. (TIF) [file pone.0031062.s004.tif]

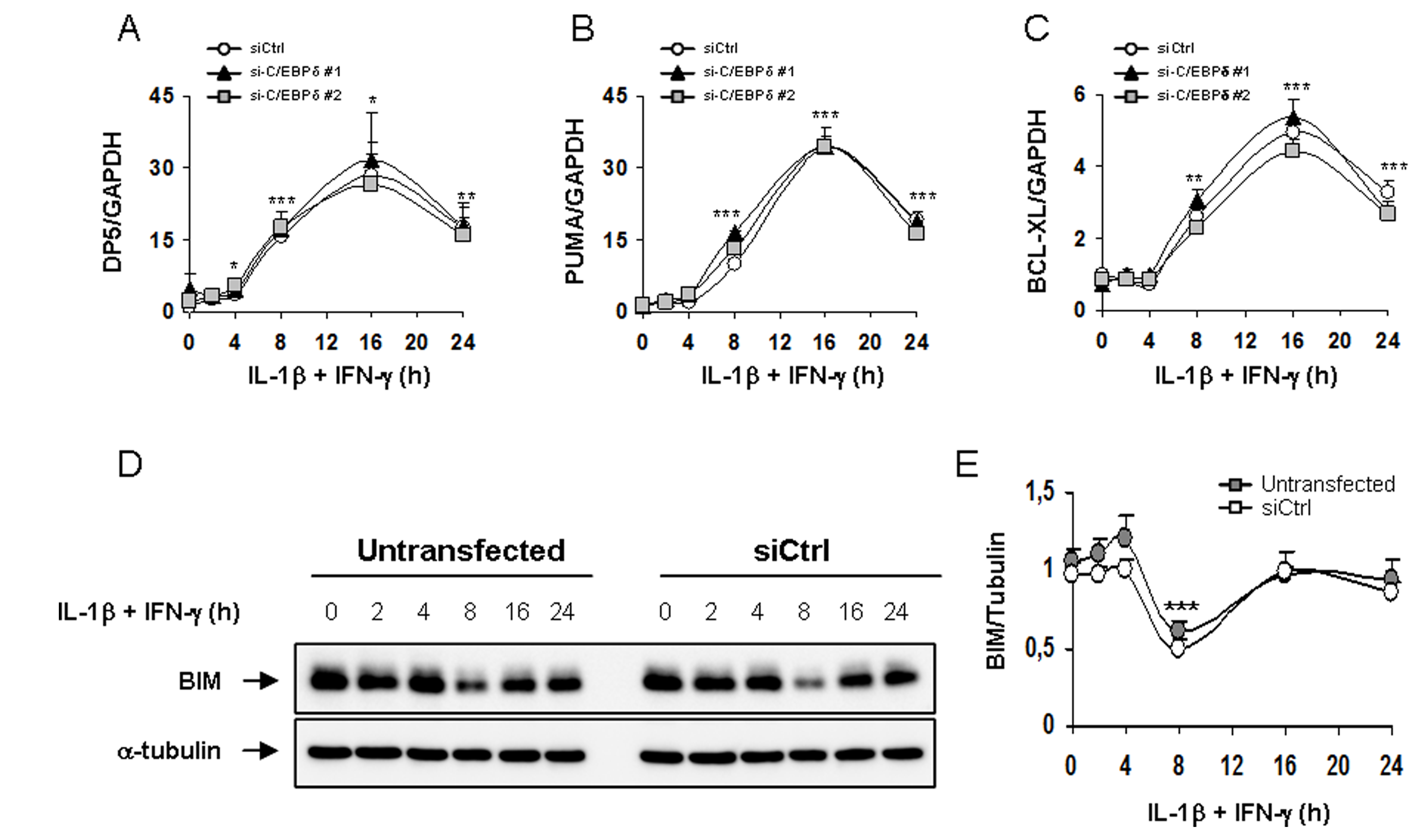

Supplement: Figure S5 — C/EBPδ knockdown doesn't affect cytokine-induced up-regulation of DP5, PUMA and Bcl-XL in INS-1E cells. (A–C) INS-1E cells were transfected with siCtrl (white dots), siC/EBPδ #1 (black triangles) or siC/EBPδ #2 (grey squares) and subsequently left untreated or treated with IL-1β+IFN-γ for the indicated time points. DP5, PUMA and Bcl-XL expressions were assayed by RT-PCR and normalized for the housekeeping gene GAPDH. (D–E) INS-1E cells were left untransfected (grey dots) or transfected with siCtrl (white dots) and subsequently left untreated or treated with IL-1β+IFN-γ for the indicated time points. (D) BIM and α-tubulin expression were evaluated by Western blot. (E) Mean optical density measurements of BIM Western blots corrected for α-tubulin (representative figure in E). Results are mean ± SEM of 4–5 independent experiments; *: p<0.05, **: p<0.01 and ***: p<0.001 vs untreated untransfected or transfected with the same siRNA; ANOVA followed by Student's t test with Bonferroni correction. (TIF) [file pone.0031062.s005.tif]

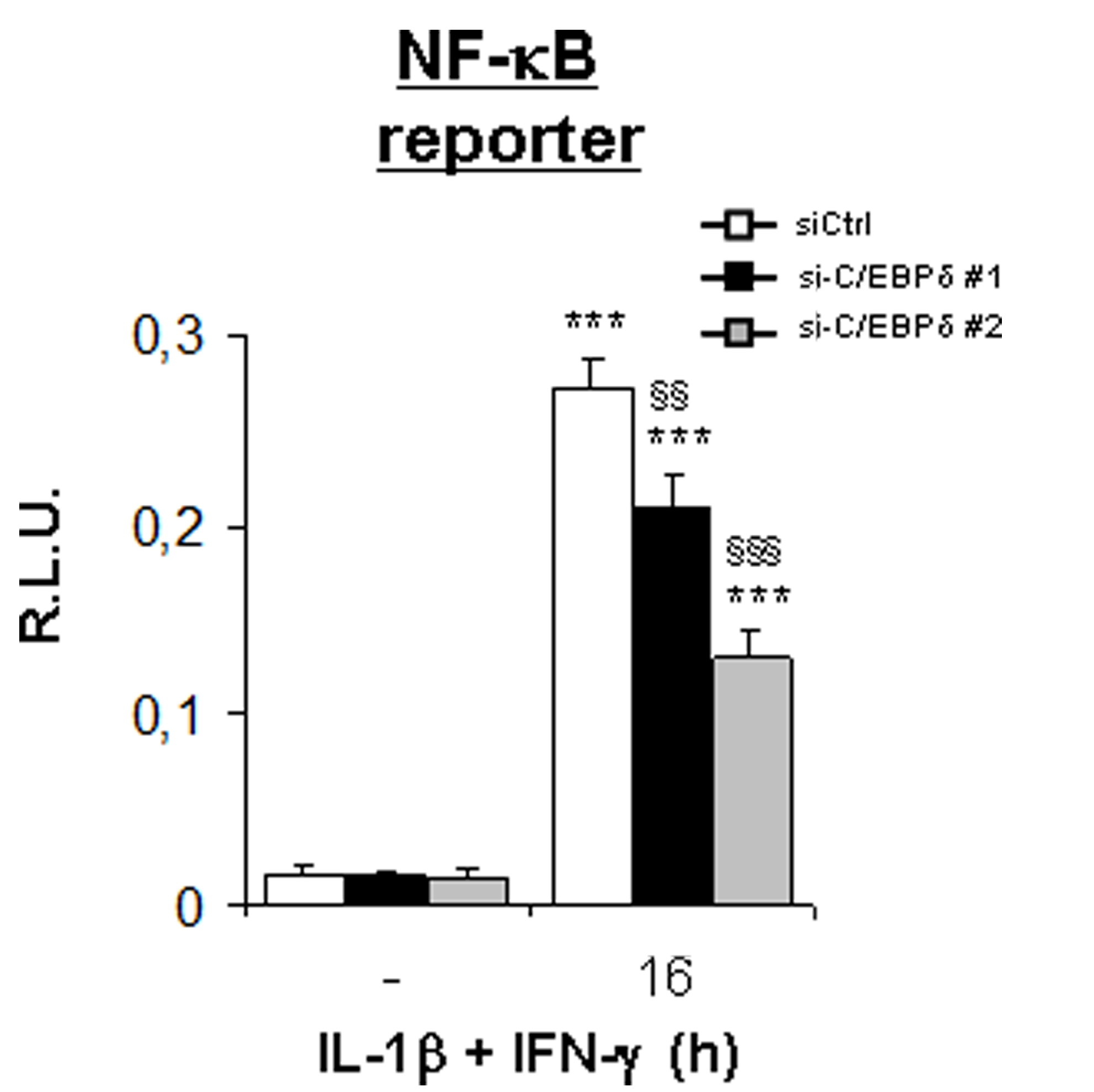

Supplement: Figure S6 — C/EBPδ silencing decreases the activation of an NF-κB luciferase reporter. INS-1E cells were transfected with siCtrl (white bars), siC/EBPδ #1 (black bars) or siC/EBPδ #2 (grey bars). After 24 h, cells were transfected with a NF-κB luciferase reporter+pRL-CMV and subsequently left untreated or exposed to IL-1β+IFN-γ for 16 h as indicated. Results are mean Relative Luciferase Unit (R.L.U.) ± SEM of 5 independent experiments; ***: p<0.001 vs untreated transfected with the same siRNA; §§: p<0.01 and §§§: p<0.001 vs siCtrl treated with cytokines at the same time point; ANOVA followed by Student's t test with Bonferroni correction. (TIF) [file pone.0031062.s006.tif]

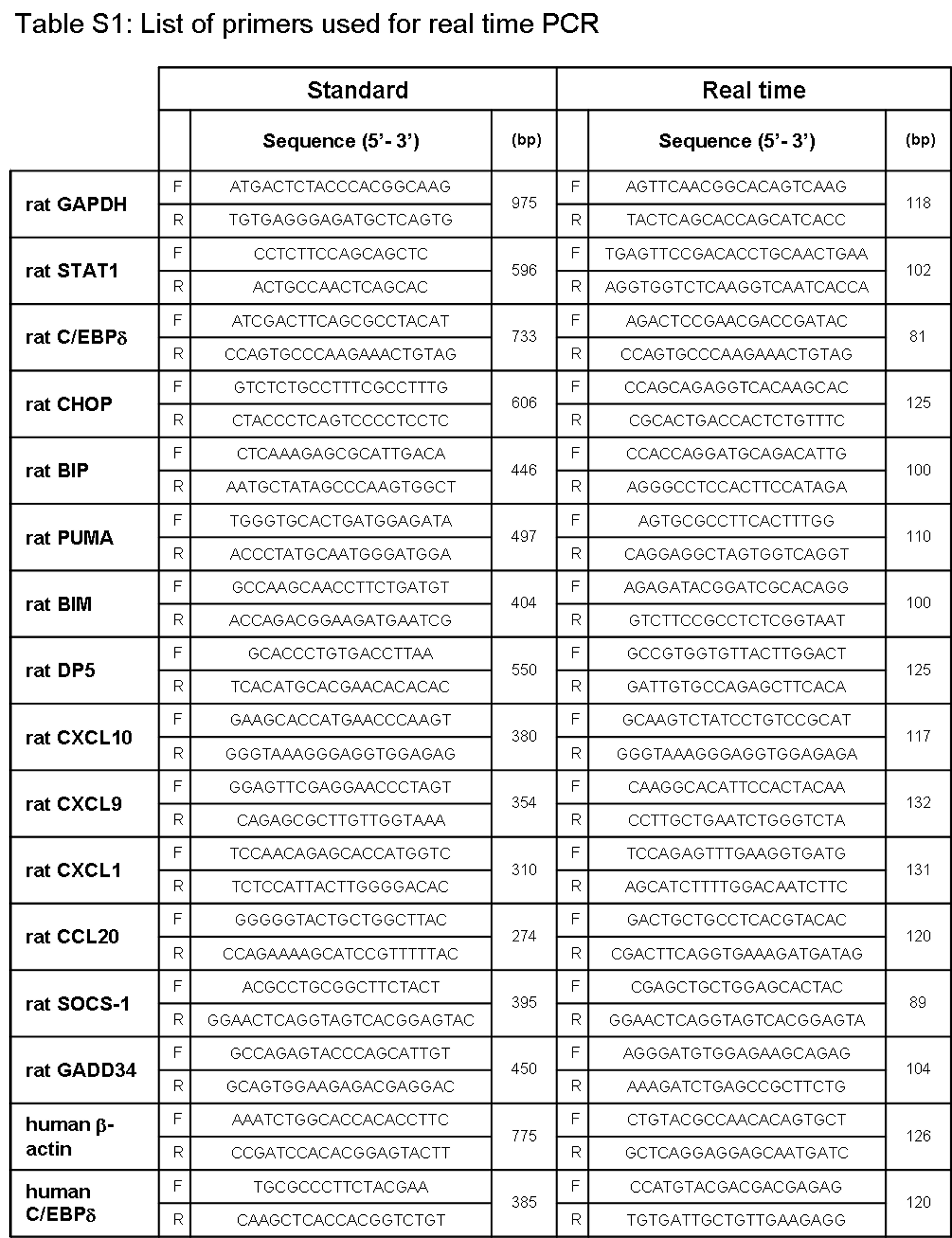

Supplement: Table S1 — List of primers used for real time PCR. (TIF) [file pone.0031062.s007.tif]
